# Pediatric Endocrinology Milestones 2.0—guide to their implementation

**DOI:** 10.1186/s12909-023-04862-5

**Published:** 2023-12-20

**Authors:** Cara V. Tillotson, Imen Becetti, Katherine Hwu, Laura Page, Sowmya Krishnan, Dianne Stafford, Takara Stanley, Patricia Vuguin, Jennifer M. Barker

**Affiliations:** 1 Pediatric Endocrinology, New York Presbyterian – Columbia University Medical Center, 622 West 168th street, PH 17 West, Room 307, New York, NY 10032 USA; 2 Pediatric Endocrinology, Massachusetts General Hospital, Boston, MA USA; 3 Pediatric Endocrinology, Baylor College of Medicine, Houston, TX USA; 4 Pediatric Endocrinology, Duke University School of Medicine, Durham, NC USA; 5 Pediatric Endocrinology, The University of Texas Southwesteren Medical Center, Dallas, TX USA; 6 Pediatric Endocrinology, Stanford University School of Medicine, Stanford, CA USA; 7 Pediatric Endocrinology, University of Colorado Anschutz Medical Campus and Children’s Hospital Colorado, Aurora, CO USA

**Keywords:** Milestones, Graduate Medical Education, Fellowship, Adult Learning

## Abstract

The Milestones were initiated by the Accreditation Council for Graduate Medical Education (ACGME) to provide a framework for monitoring a trainee’s progression throughout residency/fellowship. The Milestones describe stepwise skill progression through six core domains of clinical competency: Patient Care, Medical Knowledge, Interpersonal and Communication Skills, Practice-based Learning and Improvement, Professionalism, and Systems-based Practice. Since their introduction in 2013, several barriers to implementation have emerged. Thus, the ACGME launched the Milestones 2.0 project to develop updated specialty-specific milestones. The Pediatric Endocrinology Milestones 2.0 project aimed to improve upon Milestones 1.0 by addressing common limitations, providing resources for faculty to easily incorporate milestones into their assessment of trainees, and adding sub-competencies in health disparities, patient safety, and physician well-being.

This paper reviews the development of the Pediatric Endocrinology Milestones 2.0 including the major changes from Milestones 1.0, development of the Supplemental Guide, and how Milestones 2.0 can be applied at the program level. Although use of the Milestones are required only for ACGME programs, the tools provided in Milestones 2.0 are applicable to fellowship programs worldwide.

**Supplementary Information:**

The online version contains supplementary material available at 10.1186/s12909-023-04862-5.

## Background

The Accreditation Council for Graduate Medical Education (ACGME), jointly with the American Board of Medical Specialties (ABMS), established the six core domains of clinical competency in 1999, providing a framework for physician training and assessment of trainee progress across all specialties [1]. These core competencies include patient care (PC), medical knowledge (MK), interpersonal and communication skills (ICS), practice-based learning and improvement (PBLI), professionalism (PROF), and systems-based practice (SBP) [2].

To facilitate the integration of competencies into individual subspecialties, the Milestones were introduced in 2013 as part of the Next Accreditation System [1, 3]. For each subspecialty, milestones describe stepwise trajectories under each of the six core competencies and provide examples to guide the development of physicians in graduate medical education [4]. In addition to serving as a structure to conceptualize physician development, milestones are used to assess trainee competence and progression throughout their post-graduate clinical training.

The integration of the Milestones into training has faced limitations [3–5]. Feedback obtained by the ACGME had several common themes. First, the original Milestones were lengthy and complex, making them difficult to assess and time-consuming to complete. The multifaceted descriptors of individual milestones also made it difficult to assign levels to trainees who did not meet all characteristics. Further, the complicated language of the milestones led to variations in implementation, thus preventing a shared mental model among programs. Finally, the examples used in the sub-competencies were often not applicable across all specialties [3, 6, 7].

To address these common concerns, the ACGME launched the Milestones 2.0 project in 2016 with the goal of developing harmonized, consistent, and applicable milestones for each subspecialty [3]. The ACGME first developed cross-specialty “harmonized” milestones for ICS, PBLI, PROF, and SBP, ensuring that all specialties would now use the same descriptions for each of these milestones. To create these milestones, the ACGME reviewed feedback provided on the original milestones and data published regarding the Milestones implementation and limitations. They then created development groups of key stakeholders (content experts, directors, interprofessional team members, and other faculty) to develop unified milestones for the above domains and sub-competencies. Public comment was then invited on these milestones prior to finalization [8, 9].

The ACGME then assembled working groups for each specialty to develop specialty specific content for the medical knowledge (MK) and patient care (PC) competencies as well as a specialty specific supplemental guide. The goals of this project were to revise the Milestones to be more understandable and user-friendly while creating a shared mental model among program leadership, faculty, and fellows. In a review of shared mental models in GME, ACGME states that “a shared mental model refers to a team’s common understanding of a their task, interpretation of their environment, and required collaboration [10].^”^ Shared mental models represent one strategy for addressing some of the common limitations of the original Milestones, namely the variability among evaluators of an individual. Therefore, one of the major goals of Milestones 2.0 was to create a useable shared mental model in order to provide more consistent constructive feedback while decreasing inter-evaluator variability.

Additionally, the group aimed to include subspecialty focused skills and examples in the Milestones and exclude any sub-competencies that are irrelevant to the field of pediatric endocrinology. The supplemental guide was then developed with the goal of providing additional support for the implementation of the Milestones into practice. Finally, much of the wording in the original milestones focused on negative aspects of a fellow’s performance or indicated goals they had not yet obtained rather than highlighting their progress. Therefore, the working group aimed to reword milestones to promote a growth mindset by focusing on the skills and goals that have been reached while identifying areas for continued improvement. A “growth mindset” refers to the shared belief that learners are capable of improvement with appropriate coaching and effort [11].

## Methods

### Identification of the working group

The working group was assembled by identifying representatives from the Pediatric Endocrine Society Training Committee and soliciting applications from the community of Pediatric Endocrinologists through the ACGME, Pediatric Endocrine Society, the Association of Pediatric Program Directors, Council of Pediatric Subspecialties, and the American Board of Pediatrics. The committee comprised of twelve pediatric endocrinologists involved with pediatric endocrine education, including four current fellowship program directors, three former fellowship directors, one current associate fellowship program director, two current fellows, and two additional practicing pediatric endocrinologists with experience and interest in education and fellow assessment. Additionally, three representatives from ACGME facilitated the group’s reviews and discussions.

### Governing principles

The working group identified several governing principles for the development and assessment of the competencies and the Supplemental Guide using ACGME’s reports on the common concerns as a guide for areas of improvement. These principles included ease of interpretation, applicability to the role of a Pediatric Endocrine Fellow, and clear progression of skills across the Milestones. By using these principles, the working group aspired to produce a tool (the Milestones) that could more easily be incorporated and utilized by the busy practicing clinician while also providing valuable feedback to trainees. The working group reviewed Milestones 2.0 from other subspecialties within pediatrics and internal medicine as a model for the development of pediatric endocrine specific sub-competencies and milestones.

### Milestones development

To guide the identification of sub-competencies to be evaluated, the working group reviewed the Pediatric Subspecialty Milestones 1.0 (currently used to assess pediatric endocrinology fellows), Milestones 2.0 for Internal Medicine, Endocrinology, and Pediatrics, and drafts of Milestones 2.0 for other pediatric subspecialties. The group met virtually to identify sub-competencies appropriate for pediatric endocrinology, then convened in person to develop language for the levels for each sub-competency. ACGME representatives facilitated group discussions, during which the group reviewed the above materials and came to a consensus for each sub-competency. The Milestones were designed to allow for growth of the fellow over the course of the fellowship.

### Supplemental guide development

The working group developed a supplemental guide to aid in the interpretation of the sub-competencies and milestones as well as provide curricular opportunities to support the achievement of the milestones and potential assessment tools.

### Community feedback

Once the sub-competencies, individual milestones, and supplemental guide were developed, the draft was released to the public for comment. Comments were solicited through the Pediatric Endocrine Society, Association of Pediatric Program Directors, and the ACGME. Comments were reviewed and suggested changes to the sub-competencies and milestones were made.

## Results

The final product of the working group was the Pediatric Endocrine Milestones 2.0 and the Supplemental Guide, copies of which are included as supplemental materials. The Milestones 2.0 were officially implemented in July 2023.

### Major Changes in Milestones 2.0

Pediatric Endocrinology Milestones 2.0 brings important changes in sub-competency content as well as milestone application and usability. A major goal of Milestones 2.0 is to make the milestones more understandable and user-friendly and promote the creation of a shared mental model among program leadership, faculty, and fellows [8, 9]. This improved tool may allow fellows and faculty to track fellows’ development throughout fellowship and identify areas of strengths and weakness to be addressed early in training.

### Changes to milestone complexity and wording

Milestones 1.0 frequently included lengthy and complex descriptions making their application arduous [3]. Additionally, educational jargon made interpretations challenging for those without a strong background in educational principles. In Milestones 2.0, descriptions have been greatly shortened and jargon has been removed. Tables 1 and 2 (below) provide examples comparing the original milestones for Patient Care (PC) to their revised forms in milestones 2.0. These comparisons are examples of the simplification of the wording and the removal of the educational jargon.

**Table 1:** **a** and** b** The original Milestone Patient Care 2 (a) and 3 (b)

Level 1	Level 2	Level 3	Level 4	Level 5
**a. PC2:Make informed diagnostic and therapeutic decisions that result in optimal clinical judgement**				
Recalls and presents clinical facts in the history and physical in the order they were elicited without filtering, reorganization, or synthesis; demonstrates analytic reasoning through basic pathophysiology results in a list of all diagnoses considered rather than the development of working diagnostic considerations, making it difficult to develop a therapeutic plan	Focuses on features of the clinical presentation, making a unifying diagnosis elusive and leading to a continual search for new diagnostic possibilities; largely uses analytic reasoning through basic pathophysiology in diagnostic and therapeutic reasoning; often reorganizes clinical facts in the history and physical examination to help decide on clarifying tests to order rather than to develop and prioritize a differential diagnosis, often resulting in a myriad of tests and therapies and unclear management plans, since there is no unifying diagnosis	Abstracts and reorganizes elicited clinical findings in memory, using semantic qualifiers (such as paired opposites that are used to describe clinical information [e.g., acute and chronic]) to compare and contrast the diagnoses being considered when presenting or discussing a case; shows the emergence of pattern recognition in diagnostic and therapeutic reasoning that often results in a well-synthesized and organized assessment of the focused differential diagnosis and management plan	Reorganizes and stores clinical information (illness and instance scripts) that lead to early directed diagnostic hypothesis testing with subsequent history, physical examination, and tests used to confirm this initial schema; demonstrates well-established pattern recognition that leads to the ability to identify discriminating features between similar patients and to avoid premature closure; Selects therapies that are focused and based on a unifying diagnosis, resulting in an effective and efficient diagnostic work-up and management plan tailored to address the individual patient	Current literature does not distinguish between behaviors of proficient and expert practitioners. Expertise is not an expectation of GME training, as it requires deliberate practice over time
**b. PC3: Develop and carry out management plans**				
Develops and carries out management plans based on directives from others, either from the health care organization or the supervising physician; is unable to adjust plans based on individual patient differences or preferences; communication about the plan is unidirectional from the practitioner to the patient and family	Develops and carries out management plans based on one’s theoretical knowledge and/or directives from others; can adapt plans to the individual patient, but only within the framework of one’s own theoretical knowledge; is unable to focus on key information, so conclusions are often from arbitrary, poorly prioritized, and time-limited information gathering; develops management plans based on the framework of one’s own assumptions and values	Develops and carries out management plans based on both theoretical knowledge and some experience, especially in managing common problems; follows health care institution directives as a matter of habit and good practice rather than as an externally imposed sanction; is able to more effectively and efficiently focus on key information, but still may be limited by time and convenience; begins to incorporate patients’ assumptions and values into plans through more bidirectional communication	Develops and carries out management plans based most often on experience; effectively and efficiently focuses on key information to arrive at a plan; incorporates patients’ assumptions and values through bidirectional communication with little interference from personal biases	Develops and carries out management plans, even for complicated or rare situations, based primarily on experience that puts theoretical knowledge into context; rapidly focuses on key information to arrive at the plan and augments that with available information or seeks new information as needed; has insight into one’s own assumptions and values that allow one to filter them out and focus on the patient/family values in a bidirectional conversation about the management plan

**Table 2:** **a**, **b**, and **c** The new Patient Care 1, 2, and 3 (PC1. PC2, and PC3) of Milestones 2.0

Level 1	Level 2	Level 3	Level 4	Level 5
**a. PC1: History**				
Acquires a comprehensive and developmentally appropriate pediatric medical history Reviews available medical records	Acquires an endocrine history and a comprehensive pediatric medical history, including pubertal development and other pertinent positives and negatives Identifies relevant findings in the medical record	Acquires a tailored endocrine history, including growth, historical subtleties, and psychosocial aspects Independently requests additional information to supplement available medical records	Efficiently integrates the patient history with the complete medical record, supplemental information, and tailored assessment of potential endocrine disorders	Is identified as a peer resource in interpreting subtleties and recognizing ambiguities in the patient history
**b. PC2: Physical Exam**				
Performs a developmentally appropriate complete physical examination, with awareness of patient comfort	Performs a developmentally appropriate complete physical examination using strategies to optimize patient comfort and identifies abnormal endocrine findings	Performs a tailored physical examination using strategies to optimize patient comfort and identifies subtle abnormal endocrine findings	Detects, pursues, and integrates key physical examination findings to distinguish nuances among competing, often similar diagnoses	Is identified as a peer resource for performing tailored physical exams, maximizing patient comfort
**c. PC3: Patient Management**				
Reports and implements management plans developed by others for routine endocrine presentations	Develops and implements management plans that require modification for routine endocrine presentations	Develops and implements management plans for routine endocrine presentations	Develops and implements management plans for complex endocrine presentations, and modifies plans as necessary	Is identified as a peer resource for development of management plans for complex endocrine presentations, and modifies plans as necessary

While the total number of sub-competencies for pediatric endocrinology has increased from 21 to 24, we expect that milestone assignments should be more straightforward and therefore ideally faster to complete.

### Changes to phrasing and interpretation of milestone levels

Among the most important changes in Milestones 2.0 is how the milestone levels are phrased and applied to fellows. Milestones 1.0 created five levels based on the Dreyfus model of adult skill acquisition [12]. These levels were meant to follow trainees from novice (level 1) to expert (level 5). Because level 5 represented individuals who were experts in pediatric endocrinology, it represented an “aspirational” target that would rarely, if ever, be achieved by fellows. It was also unclear if fellows should “reset” to level 1 after achieving 3 s and 4 s in a particular milestone at the time of residency graduation.

Milestone 2.0 intends to document developmental progression during fellowship, rather than the entire training or career trajectory. Thus, it is expected that many fellows will enter fellowship at a level 1 (novice fellow) and subsequently progress at varying rates to level 2 (advanced beginner), level 3 (competent) and level 4 (proficient). This is especially true for the patient care (PC) and medical knowledge (MK) competencies, which are now specific to pediatric endocrinology. While not a graduation requirement, it is expected that most fellows will achieve a level 4 in most milestones prior to graduation. Level 5 now represents an expert fellow, corresponding to a fellow performing exceptionally in a given sub-competency. For a given sub-competency, ACGME provides guidance that appropriately 8–10% of fellows should achieve a level 5 prior to graduation.

The phrasing of Milestones 2.0 has also been changed to promote a growth mindset. Milestones 1.0 frequently used negative language that emphasized skills that a fellow was not doing or was doing incorrectly. Milestones 2.0 focuses on what the fellow is correctly doing at each developmental stage. For example, Milestones 1.0, sub-competency PM, Level 2 includes the description “is unable to focus on key information so conclusions are often from arbitrary, poorly prioritized, and time-limited information gathering.” In Milestones 2.0, Level 2 of the same sub-competency reads “Develops and implements management plans that require modification for routine endocrine presentations.” It is recognized that some fellows may not yet have achieved level 1 when they enter fellowship. Therefore, Milestones 2.0 retains the option of selecting “not yet completed level 1” for assessments.

### Creation of harmonized milestones

A major change for Milestones 2.0 is the creation of “harmonized” milestones in four competencies: Professionalism (PROF), Practice-based learning and improvement (PBLI), Interpersonal and communication skills (ICS), and Systems-based practice (SBP). In Milestones 1.0, specialties created their own content for each competency, leading to highly variable themes and descriptions [7]. These inconsistencies created challenges in comparing milestone progression among specialties and in sharing learning tools and resources. Having recognized that PROF, PBLI, ICS, and SBP have common, overlapping themes for most specialties, the ACGME assembled four diverse groups to develop cross-specialty “harmonized” milestones for Milestones 2.0 [9]. Pediatric Endocrinology Milestones 2.0 adopts harmonized milestones in each of these four competencies.

### Creation of milestones specific to pediatric endocrinology

Pediatric Endocrinology Milestones 2.0 modifies the sub-competencies and milestones for Patient Care (PC) and Medical Knowledge (MK) to be more tailored to pediatric endocrinology. The changes to the PC sub-competency milestones are outlined in Table 3 below.

**Table 3:** Comparison of the PC sub-competencies between Milestones 1.0 and 2.0

Patient Care (PC) Changes	
**Milestones 1.0**	**Milestones 2.0**
**PC1:** Provide transfer of care that ensures seamless transitions	**PC1:** History
**PC2:** Make informed diagnostic and therapeutic decisions that result in optimal clinical judgement	**PC 2:** Physical Exam
**PC3:** Develop and carry out management plan	**PC3:** Patient Management
**PC4:** Provide appropriate role modeling	**PC4:** Diagnostic testing (including labs, imaging, and functional testing)
	**PC5:** Clinical Consultation

Milestones 1.0 included PC1: Provide transfer of care that ensures seamless transitions; PC2: Make informed diagnostic and therapeutic decisions that result in optimal clinical judgment; PC3: Develop and carry out management plan and PC4. Provide appropriate role modeling. In Milestones 2.0 this was changed to PC1: History; PC2: Physical Exam; PC3: Patient Management; PC4: Diagnostic Testing (including Labs, imaging, and functional testing) and PC5: Clinical Consultation. Each of these sub-competencies then had milestone language specific to pediatric endocrine fellowship training with the supplementary guide illustrating examples in a particular scenario. A fifth PC sub-competency of consultation was added as it was felt to be a core skill acquired during fellowship training. Many other subspecialty Milestones 2.0 incorporate the consultation sub-competency, including the adult endocrinology Milestones.

Table 4  (below) outlines the changes to the MK sub-competencies. Milestones 1.0 was limited to MK1: locate, appraise, and assimilate evidence from scientific studies related to their patients’ health problems. The milestone language under this sub-competency was long and multipronged, making assessments challenging. In Milestones 2.0 MK is separated into three sub-competencies: MK1: Physiology and Pathophysiology, MK2: Clinical Reasoning; MK3: Therapeutics (Behavioral, Medications, Technology, Radiopharmaceuticals). This change intuitively makes more sense and will allow fellows and faculty to identify specific areas of strength and need for improvement.

**Table 4:** Comparison of the MK sub-competencies between Milestones 1.0 and 2.0

Medical Knowledge (MK) Changes	
**Milestones 1.0**	**Milestones 2.0**
**MK1:** locate, appraise, and assimilate evidence from scientific studies related to patients’ health conditions	**MK1:** Physiology and Pathophysiology
	**MK2:** Clinical Reasoning
	**MK3:** Therapeutics (behavioral, medications, technology, radiopharmaceuticals)

### New sub-competency concepts

Milestones 2.0 addresses several topics that were not emphasized in Milestones 1.0. SBP now includes a specific sub-competency on population and community health that incorporates the concept of health disparities. A separate SBP sub-competency focuses on patient safety, which was previously combined with medical errors and inter-professional teamwork in Milestones 1.0. Additionally, a new PROF sub-competency centers on the concept of well-being. This sub-competency does not evaluate a fellow’s personal well-being but instead recognizes and emphasizes the importance of understanding factors that affect fellow and physician well-being [13].

### Creation of the supplemental guide

Finally, an important addition to Milestones 2.0 is the creation of a Supplemental Guide that clarifies the intentions of the working group for each milestone. The guide will be available for program directors, clinical competency committees (CCCs), and fellows to promote a shared mental model, which is one of the primary goals of Milestones 2.0. The Supplemental Guide is available as a word document as well as PDF so that individual programs can edit the guide to make it more meaningful to their program.

The Supplemental Guide includes five sections for each sub-competency: 1) the overall intent for the sub-competency, 2) a general example for each level, 3) suggested assessment tools to be used by programs in determining level, 4) curriculum mapping (left blank as it is to be completed by the individual program), and 5) notes or resources. The examples included for each level are not comprehensive nor are they indicative of a specific requirement. Instead, the examples are a conversation starting point in creating the shared mental model.

### What is the same

Despite many changes with Milestones 2.0, some key concepts remain the same. Fellows should be assigned a milestone level that fits their current performance, regardless of their year in fellowship. A fellow should have met the criteria of their assigned level and those of the preceding level(s). The ACGME has no level requirement that a fellow must achieve to graduate. Instead, graduation readiness is determined by the fellow’s program director, scholarly oversight committee, and CCC [12]. Similarly, Milestones 2.0 is not part of the endocrinology certification eligibility requirements established by the American Board of Pediatrics (ABP) and milestone levels are not reported to the ABP. Finally, the milestone set is intended to monitor fellow progression over extended periods of time. Therefore, it has limited utility in short rotations of 2–8 weeks [6].

## Discussion

### Ways to implement milestones into practice

The new ACGME common program requirements state that milestones are to be incorporated into the semiannual evaluation process. Following determination by the CCC, fellows should receive feedback on milestone levels as these may be useful to identify areas of strength and weakness and to establish learning plans.

Milestones can also be utilized for fellow self-assessment or to monitor the areas for improvement in a program. Programs may choose to have fellows complete a self-assessment of milestone levels each time the CCC is going to meet. The program director and fellow can then compare both sets of assessment which may be helpful for both the program and the resident. The program will have insight into the fellow’s understanding of their knowledge skills and attitudes and the fellow will be able to calibrate their own awareness. Similarly, CCCs can review the milestones of all of their fellows to determine if there are common areas in which their trainees are not progressing as expected, which could represent areas in which their fellowship should focus on improving education.

## Conclusion

The Milestones were developed to be an important tool in career progression of trainees, but implementation has been hindered by being overly complex and burdensome. The new pediatric endocrine Milestones 2.0 and the supplemental guide are intended to make the milestones more applicable to our field, easier to utilize, focused on individual growth, and more attentive to important issues of health equity and population health. Further research and feedback on the Milestones 2.0 after implementation will determine whether these goals were met. While the Milestones are required only in fellowships accredited by the ACGME, their general principles are applicable to trainees worldwide and can be another tool in the evaluation of a fellow’s progress through their career.

### Supplementary Information

**Additional file 1.**

**Additional file 2. **

## Data Availability

Not applicable.
