# Supplementary material for: Pediatric Endocrinology Milestones 2.0—guide to their implementation
Source: BMC Med Educ. 2023 Dec 20;23:985. doi: 10.1186/s12909-023-04862-5 (PMC10734147; doi:10.1186/s12909-023-04862-5)
Supplement: Supplementary file 1 — Additional file 1. [file 12909_2023_4862_MOESM1_ESM.pdf]

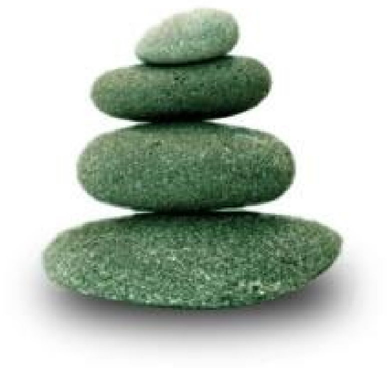

Pediatric Endocrinology Milestones

The Accreditation Council for Graduate Medical Education

ACGME

Implementation Date: July 2023

Second Revision: April 2023
First Revision: January 2014

©2022 Accreditation Council for Graduate Medical Education (ACGME)
All rights reserved except the copyright owners grant third parties the right to use the Pediatric Endocrinology Milestones on a non-exclusive basis for educational purposes.

Pediatric Endocrinology Milestones

The Milestones are designed only for use in evaluation of fellows in the context of their participation in ACGME-accredited residency or fellowship programs. The Milestones provide a framework for the assessment of the development of the fellow in key dimensions of the elements of physician competency in a specialty or subspecialty. They neither represent the entirety of the dimensions of the six domains of physician competency, nor are they designed to be relevant in any other context.

©2022 Accreditation Council for Graduate Medical Education (ACGME)
All rights reserved except the copyright owners grant third parties the right to use the Pediatric Endocrinology Milestones on a non-exclusive basis for educational purposes.

Pediatric Endocrinology Milestones Work Group

Jennifer Barker, MD

Imen Becetti, MD

Laura Edgar, EdD, CAE

Robert Hoffman, MD

Michele Hutchison, MD, PhD

Katherine Hwu, MD

Sowmya Krishnan, MD

Juan Lado, MD

Laura Page, MD

Diane Stafford, MD

Takara Stanley

Cara Tillotson, DO

Patricia Vuguin, MD, MS

The ACGME would like to thank the following organizations for their continued support in the development of the Milestones:

American Board of Pediatrics

Association of Pediatric Program Directors

Council of Pediatric Subspecialties

ACGME Review Committee for Pediatrics

©2022 Accreditation Council for Graduate Medical Education (ACGME)
All rights reserved except the copyright owners grant third parties the right to use the Pediatric Endocrinology Milestones on a non-exclusive basis for educational purposes.

Understanding Milestone Levels and Reporting

This document presents the Milestones, which programs use in a semi-annual review of fellow performance, and then report to the ACGME. Milestones are knowledge, skills, attitudes, and other attributes for each of the ACGME Competencies organized in a developmental framework. The narrative descriptions are targets for fellows' performance throughout their educational program.

Milestones are arranged into levels. Tracking from Level 1 to Level 5 is synonymous with moving from novice to expert fellow in the specialty or subspecialty. For each reporting period, the Clinical Competency Committee will review the completed evaluations to select the milestone levels that best describe each learner's current performance, abilities, and attributes for each subcompetency.

These levels *do not* correspond with post-graduate year of education. Depending on previous experience, a junior fellow may achieve higher levels early in the educational program just as a senior fellow may be at a lower level later in the educational program. There is no predetermined timing for a fellow to attain any particular level. Fellows may also regress in achievement of their milestones. This may happen for many reasons, such as over scoring in a previous review, a disjointed experience in a particular procedure, or a significant act by the fellow.

Selection of a level implies the fellow substantially demonstrates the milestones in that level, as well as those in lower levels (see the diagram on page vi).

©2022 Accreditation Council for Graduate Medical Education (ACGME)
All rights reserved except the copyright owners grant third parties the right to use the Pediatric Endocrinology Milestones on a non-exclusive basis for educational purposes.

Additional Notes

Level 4 is designed as a graduation *goal* but *does not* represent a graduation *requirement*. Making decisions about readiness for graduation and unsupervised practice is the purview of the program director. Furthermore, Milestones 2.0 include revisions and changes that preclude using Milestones as a sole assessment in high-stakes decisions (i.e., determination of eligibility for certification or credentialing). Level 5 is designed to represent an expert fellow whose achievements in a subcompetency are greater than the expectation. Milestones are primarily designed for formative, developmental purposes to support continuous quality improvement for individual learners, education programs, and the specialty. The ACGME and its partners will continue to evaluate and perform research on the Milestones to assess their impact and value.

Examples are provided for some milestones within this document. Please note: the examples are not the required element or outcome; they are provided as a way to share the intent of the element.

A Supplemental Guide is also available to provide the intent of each subcompetency, examples for each level, assessment methods or tools, and other available resources. The Supplemental Guide, like examples contained within the Milestones, is designed only to assist the program director and Clinical Competency Committee, and is not meant to demonstrate any required element or outcome.

Additional resources are available in the Milestones section of the ACGME website. Follow the links under "What We Do" at www.acgme.org.

©2022 Accreditation Council for Graduate Medical Education (ACGME)
All rights reserved except the copyright owners grant third parties the right to use the Pediatric Endocrinology Milestones on a non-exclusive basis for educational purposes.

The diagram below presents an example set of milestones for one sub-competency in the same format as the ACGME Report Worksheet. For each reporting period, a fellow's performance on the milestones for each sub-competency will be indicated by selecting the level of milestones that best describes that fellow's performance in relation to those milestones.

Systems-Based Practice 1: Patient Safety

Level 1	Level 2	Level 3	Level 4	Level 5
Demonstrates knowledge of common patient safety events	Identifies system factors that lead to patient safety events	Participates in analysis of patient safety events (simulated or actual)	Conducts analysis of patient safety events and offers error prevention strategies (simulated or actual)	Actively engages teams and processes to modify systems to prevent patient safety events
Demonstrates knowledge of how to report patient safety events	Reports patient safety events through institutional reporting systems (simulated or actual)	Participates in disclosure of patient safety events to patients and patients' families (simulated or actual)	Discloses patient safety events to patients and patients' families (simulated or actual)	Role models or mentors others in the disclosure of patient safety events

Comments:

Not Yet Completed Level 1

Selecting a response box in the middle of a level implies that milestones in that level and in lower levels have been substantially demonstrated.

Selecting a response box on the line in between levels indicates that milestones in lower levels have been substantially demonstrated as well as **some** milestones in the higher level(s).

©2022 Accreditation Council for Graduate Medical Education (ACGME)
All rights reserved except the copyright owners grant third parties the right to use the Pediatric Endocrinology Milestones on a non-exclusive basis for educational purposes.

Patient Care 1: History				
Level 1	Level 2	Level 3	Level 4	Level 5
Acquires a comprehensive and developmentally appropriate pediatric medical history<br><br>Reviews available medical records	Acquires an endocrine history and a comprehensive pediatric medical history, including pubertal development and other pertinent positives and negatives<br><br>Identifies relevant findings in the medical record	Acquires a tailored endocrine history, including growth, historical subtleties, and psychosocial aspects<br><br>Independently requests additional information to supplement available medical records	Efficiently integrates the patient history with the complete medical record, supplemental information, and tailored assessment of potential endocrine disorders	Is identified as a peer resource in interpreting subtleties and recognizing ambiguities in the patient history

Comments:

Not Yet Completed Level 1

Not Yet Assessable

©2022 Accreditation Council for Graduate Medical Education (ACGME)
All rights reserved except the copyright owners grant third parties the right to use the Pediatric Endocrinology Milestones on a non-exclusive basis for educational purposes.

Patient Care 2: Physical Exam				
Level 1	Level 2	Level 3	Level 4	Level 5
Performs a developmentally appropriate complete physical examination, with awareness of patient comfort	Performs a developmentally appropriate complete physical examination using strategies to optimize patient comfort and identifies abnormal endocrine findings	Performs a tailored physical examination using strategies to optimize patient comfort and identifies subtle abnormal endocrine findings	Detects, pursues, and integrates key physical examination findings to distinguish nuances among competing, often similar diagnoses	Is identified as a peer resource for performing tailored physical exams, maximizing patient comfort

Comments:

Not Yet Completed Level 1

Not Yet Assessable

©2022 Accreditation Council for Graduate Medical Education (ACGME)
All rights reserved except the copyright owners grant third parties the right to use the Pediatric Endocrinology Milestones on a non-exclusive basis for educational purposes.

**Patient Care 3: Patient Management**				
Level 1	Level 2	Level 3	Level 4	Level 5
Reports and implements management plans developed by others for routine endocrine presentations	Develops and implements management plans that require modification for routine endocrine presentations	Develops and implements management plans for routine endocrine presentations	Develops and implements management plans for complex endocrine presentations, and modifies plans as necessary	Is identified as a peer resource for development of management plans for complex endocrine presentations, and modifies plans as necessary

Comments:

Not Yet Completed Level 1

Not Yet Assessable

©2022 Accreditation Council for Graduate Medical Education (ACGME)
All rights reserved except the copyright owners grant third parties the right to use the Pediatric Endocrinology Milestones on a non-exclusive basis for educational purposes.

Patient Care 4: Diagnostic Testing, Including Labs, Imaging, and Functional Testing				
Level 1	Level 2	Level 3	Level 4	Level 5
Orders non-targeted tests for patients with routine endocrine presentations<br><br>Interprets basic endocrine test results, with guidance	Orders targeted tests for patients with routine endocrine presentations<br><br>Independently interprets targeted test results for routine endocrine presentations	Orders targeted tests for patients with complex endocrine presentations<br><br>Interprets targeted test results for patients with complex endocrine presentations, with assistance, and identifies incongruencies	Develops individualized cost-effective testing strategies to evaluate patients with complex endocrine presentations and avoids unnecessary testing<br><br>Resolves incongruencies and accepts ambiguity in targeted test results for patients with complex endocrine presentations	Identifies, critically evaluates, and selectively uses emerging and investigational tests or procedures; questions and reports unknown and unexplained discrepancies

Comments:

Not Yet Completed Level 1

Not Yet Assessable

©2022 Accreditation Council for Graduate Medical Education (ACGME)
All rights reserved except the copyright owners grant third parties the right to use the Pediatric Endocrinology Milestones on a non-exclusive basis for educational purposes.

**Patient Care 5: Clinical Consultation**				
Level 1	Level 2	Level 3	Level 4	Level 5
Responds to consultation after receiving assistance	Clarifies the clinical questions and provides preliminary recommendations to the requesting practitioner	Seeks and integrates input from different members of the health care team and provides recommendations to the requesting practitioner in a clear and timely manner	Provides comprehensive and prioritized recommendations, including assessment, rationale, and anticipatory guidance to all relevant health care team members	Is identified as a peer resource for the provision of consultative care across the spectrum of disease complexity and acuity
Recognizes disease acuity, with supervision	Independently recognizes disease acuity	Recognizes disease acuity and prioritizes management steps	Mobilizes resources based on acuity of the situation	

Comments:

Not Yet Completed Level 1
Not Yet Assessable

©2022 Accreditation Council for Graduate Medical Education (ACGME)
All rights reserved except the copyright owners grant third parties the right to use the Pediatric Endocrinology Milestones on a non-exclusive basis for educational purposes.

Medical Knowledge 1: Physiology and Pathophysiology				
Level 1	Level 2	Level 3	Level 4	Level 5
Demonstrates generalized knowledge of physiological and pathophysiological concepts in endocrinology	Demonstrates knowledge of physiology and pathophysiology of routine endocrine conditions	Applies knowledge of physiology and pathophysiology to diagnosis and management of routine presentations	Applies knowledge of physiology and pathophysiology to diagnosis and management of complex presentations	Synthesizes newly described and emerging clinical physiology and pathophysiology concepts with diagnosis and management

Comments:

Not Yet Completed Level 1
Not Yet Assessable

©2022 Accreditation Council for Graduate Medical Education (ACGME)
All rights reserved except the copyright owners grant third parties the right to use the Pediatric Endocrinology Milestones on a non-exclusive basis for educational purposes.

Medical Knowledge 2: Clinical Reasoning				
Level 1	Level 2	Level 3	Level 4	Level 5
Organizes and accurately summarizes information obtained from the patient evaluation to develop a clinical impression	Integrates information from all sources to develop a basic differential diagnosis for routine endocrine presentations<br><br>Identifies clinical reasoning errors within patient care, with guidance	Develops a thorough and prioritized differential diagnosis for routine endocrine presentations<br><br>Retrospectively applies clinical reasoning principles to identify errors	Synthesizes subtle, unusual, or conflicting findings to prioritize differential diagnoses in complex endocrine presentations<br><br>Continually re-appraises own clinical reasoning to improve patient care in real time	Coaches others to develop prioritized differential diagnoses in complex endocrine presentations<br><br>Models how to recognize errors and reflect upon one's own clinical reasoning

Comments:

Not Yet Completed Level 1

Not Yet Assessable

©2022 Accreditation Council for Graduate Medical Education (ACGME)
All rights reserved except the copyright owners grant third parties the right to use the Pediatric Endocrinology Milestones on a non-exclusive basis for educational purposes.

Medical Knowledge 3: Therapeutics (Behavioral, Medications, Technology, Radiopharmaceuticals)				
Level 1	Level 2	Level 3	Level 4	Level 5
Demonstrates knowledge of basic endocrine therapeutics	Demonstrates knowledge of the indications, monitoring parameters, and adverse effects of endocrine therapeutics	Applies knowledge of therapeutics to the management of patients with routine endocrine conditions	Applies knowledge of therapeutics to the management of patients with complex endocrine conditions	Identifies targeted or experimental therapies for complex and rare clinical scenarios

Comments:

Not Yet Completed Level 1
Not Yet Assessable

©2022 Accreditation Council for Graduate Medical Education (ACGME)
All rights reserved except the copyright owners grant third parties the right to use the Pediatric Endocrinology Milestones on a non-exclusive basis for educational purposes.

Systems-Based Practice 1: Patient Safety				
Level 1	Level 2	Level 3	Level 4	Level 5
Demonstrates knowledge of common patient safety events	Identifies system factors that lead to patient safety events	Participates in analysis of patient safety events (simulated or actual)	Conducts analysis of patient safety events and offers error prevention strategies (simulated or actual)	Actively engages teams and processes to modify systems to prevent patient safety events
Demonstrates knowledge of how to report patient safety events	Reports patient safety events through institutional reporting systems (simulated or actual)	Participates in disclosure of patient safety events to patients and families (simulated or actual)	Discloses patient safety events to patients and families (simulated or actual)	Role models or mentors others in the disclosure of patient safety events

Comments:

Not Yet Completed Level 1

©2022 Accreditation Council for Graduate Medical Education (ACGME)
All rights reserved except the copyright owners grant third parties the right to use the Pediatric Endocrinology Milestones on a non-exclusive basis for educational purposes.

Systems-Based Practice 2: Quality Improvement				
Level 1	Level 2	Level 3	Level 4	Level 5
Demonstrates knowledge of basic quality improvement methodologies and metrics	Describes local quality improvement initiatives (e.g., insulin management, screening for diabetes complications)	Participates in local quality improvement initiatives	Demonstrates the skills required to identify, develop, implement, and analyze a quality improvement project	Creates, implements, and assesses quality improvement initiatives at the institutional or community level
☐ ☐	☐ ☐	☐ ☐	☐ ☐	☐

Comments:

Not Yet Completed Level 1 ☐

©2022 Accreditation Council for Graduate Medical Education (ACGME)
All rights reserved except the copyright owners grant third parties the right to use the Pediatric Endocrinology Milestones on a non-exclusive basis for educational purposes.

Systems-Based Practice 3: System Navigation for Patient Centered Care – Coordination of Care				
Level 1	Level 2	Level 3	Level 4	Level 5
Lists the various interprofessional individuals involved in the patient's care coordination	Coordinates care of patients in routine clinical situations, incorporating interprofessional teams with consideration of patient and family needs	Coordinates care of patients in complex clinical situations, effectively utilizing the roles of interprofessional teams, and incorporating patient and family needs and goals	Coordinates interprofessional, patient-centered care among different disciplines and specialties, actively assisting families in navigating the health care system	Coaches others in interprofessional, patient-centered care coordination

Comments:

Not Yet Completed Level 1

©2022 Accreditation Council for Graduate Medical Education (ACGME)
All rights reserved except the copyright owners grant third parties the right to use the Pediatric Endocrinology Milestones on a non-exclusive basis for educational purposes.

**Systems-Based Practice 4: System Navigation for Patient-Centered Care – Transitions in Care**				
Level 1	Level 2	Level 3	Level 4	Level 5
Uses a standard template for transitions of care/hand-offs	Adapts a standard template, recognizing key elements for safe and effective transitions of care/hand-offs in routine clinical situations	Performs safe and effective transitions of care/hand-offs in complex clinical situations, and ensures closed-loop communication	Performs and advocates for safe and effective transitions of care/hand-offs within and across health care delivery systems, including transitions to adult care	Coaches others in improving transitions of care within and across health care delivery systems to optimize patient outcomes

Comments:

Not Yet Completed Level 1

©2022 Accreditation Council for Graduate Medical Education (ACGME)
All rights reserved except the copyright owners grant third parties the right to use the Pediatric Endocrinology Milestones on a non-exclusive basis for educational purposes.

Systems-Based Practice 5: Population and Community Health				
Level 1	Level 2	Level 3	Level 4	Level 5
Demonstrates awareness of population and community health needs and disparities	Identifies specific population and community health needs and disparities; identifies local resources	Uses local resources effectively to meet the needs and reduce health disparities of a patient population and community	Adapts practice to provide for the needs of and reduce health disparities of a specific population	Advocates at the local, regional, or national level for populations and communities with health care disparities

Comments:

Not Yet Completed Level 1

©2022 Accreditation Council for Graduate Medical Education (ACGME)
All rights reserved except the copyright owners grant third parties the right to use the Pediatric Endocrinology Milestones on a non-exclusive basis for educational purposes.

Systems-Based Practice 6: Physician Role in Health Care Systems				
Level 1	Level 2	Level 3	Level 4	Level 5
Engages with patients and other providers in discussions about cost-conscious care and key components of the health care delivery system	Identifies the relationships between the delivery system and cost-conscious care and the impact on the patient care	Discusses the need for changes in clinical approaches based on evidence, outcomes, and cost-effectiveness to improve care for patients and families	Advocates for the promotion of safe, quality, and high-value care	Coaches others to promote safe, quality, and high-value care across health care systems

Comments:

Not Yet Completed Level 1

©2022 Accreditation Council for Graduate Medical Education (ACGME)
All rights reserved except the copyright owners grant third parties the right to use the Pediatric Endocrinology Milestones on a non-exclusive basis for educational purposes.

**Practice-Based Learning and Improvement 1: Evidence-Based and Informed Practice**				
Level 1	Level 2	Level 3	Level 4	Level 5
Develops an answerable clinical question and demonstrates how to access available evidence, with guidance	Independently articulates clinical question and accesses available evidence	Locates and applies the evidence, integrated with patient preference, to the care of patients	Critically appraises and applies evidence, even in the face of uncertainty and conflicting evidence to guide care tailored to the individual patient	Coaches others to critically appraise and apply evidence for complex patients

Comments:

Not Yet Completed Level 1

©2022 Accreditation Council for Graduate Medical Education (ACGME)
All rights reserved except the copyright owners grant third parties the right to use the Pediatric Endocrinology Milestones on a non-exclusive basis for educational purposes.

Practice-Based Learning and Improvement 2: Reflective Practice and Commitment to Personal Growth				
Level 1	Level 2	Level 3	Level 4	Level 5
Participates in feedback sessions	Demonstrates openness to feedback and performance data	Seeks and incorporates feedback and performance data episodically	Seeks and incorporates feedback and performance data consistently	Role models and coaches others in seeking and incorporating feedback and performance data
Develops personal and professional goals, with assistance	Designs a learning plan based on established goals, feedback, and performance data, with assistance	Designs and implements a learning plan by analyzing and reflecting on the factors which contribute to gap(s) between performance expectations and actual performance	Adapts a learning plan using long-term professional goals, self-reflection, and performance data to measure its effectiveness	Demonstrates continuous self-reflection and coaching of others on reflective practice

Comments:

Not Yet Completed Level 1

©2022 Accreditation Council for Graduate Medical Education (ACGME)
All rights reserved except the copyright owners grant third parties the right to use the Pediatric Endocrinology Milestones on a non-exclusive basis for educational purposes.

**Professionalism 1: Professional Behavior**				
Level 1	Level 2	Level 3	Level 4	Level 5
Identifies expected professional behaviors and potential triggers for lapses	Demonstrates professional behavior with occasional lapses	Maintains professional behavior in increasingly complex or stressful situations	Recognizes situations that may trigger professionalism lapses and intervenes to prevent lapses in self and others	Models professional behavior and coaches others when their behavior fails to meet professional expectations
Identifies the value and role of pediatric endocrinology as a vocation/career	Demonstrates accountability for patient care as a pediatric endocrinologist, with guidance	Fully engages in patient care and holds oneself accountable	Exhibits a sense of duty to patient care and professional responsibilities	Extends the role of the pediatric endocrinologist beyond the care of patients by engaging with the community, specialty, and medical profession as a whole

Comments:

Not Yet Completed Level 1

©2022 Accreditation Council for Graduate Medical Education (ACGME)
All rights reserved except the copyright owners grant third parties the right to use the Pediatric Endocrinology Milestones on a non-exclusive basis for educational purposes.

Professionalism 2: Ethical Principles				
Level 1	Level 2	Level 3	Level 4	Level 5
Demonstrates knowledge of the ethical principles underlying informed consent, surrogate decision making, advance directives, confidentiality, error disclosure, stewardship of limited resources, and related topics	Applies ethical principles in common situations	Analyzes complex situations using ethical principles to address conflict/controversy; seeks help when needed to manage and resolve complex ethical situations	Manages and seeks to resolve ethical dilemmas using appropriate resources (e.g., ethics consultations, literature review, risk management/legal consultation)	Called upon by others to consult in cases of complex ethical dilemmas; identifies and seeks to address system-level factors that induce or exacerbate

Comments:

Not Yet Completed Level 1

©2022 Accreditation Council for Graduate Medical Education (ACGME)
All rights reserved except the copyright owners grant third parties the right to use the Pediatric Endocrinology Milestones on a non-exclusive basis for educational purposes.

**Professionalism 3: Accountability/Conscientiousness**				
Level 1	Level 2	Level 3	Level 4	Level 5
Performs tasks and responsibilities, with prompting	Performs tasks and responsibilities in a timely manner in routine situations	Performs tasks and responsibilities in a thorough and timely manner in complex or stressful situations	Coaches others to ensure tasks and responsibilities are completed in a thorough and timely manner in complex or stressful situations	Creates strategies to enhance others' ability to efficiently complete tasks and responsibilities

Comments:

Not Yet Completed Level 1

©2022 Accreditation Council for Graduate Medical Education (ACGME)
All rights reserved except the copyright owners grant third parties the right to use the Pediatric Endocrinology Milestones on a non-exclusive basis for educational purposes.

Professionalism 4: Well-Being				
Level 1	Level 2	Level 3	Level 4	Level 5
Recognizes the importance of addressing personal and professional well-being	Describes institutional resources that are meant to promote well-being	Recognizes institutional and personal factors that impact well-being	Describes interactions between institutional and personal factors that impact well-being	Coaches and supports colleagues to optimize well-being at the team, program, or institutional level

Comments:

Not Yet Completed Level 1

This subcompetency is not intended to evaluate a fellow's well-being, but to ensure each fellow has the fundamental knowledge of factors that impact well-being, the mechanisms by which those factors impact well-being, and available resources and tools to improve well-being.

©2022 Accreditation Council for Graduate Medical Education (ACGME)
All rights reserved except the copyright owners grant third parties the right to use the Pediatric Endocrinology Milestones on a non-exclusive basis for educational purposes.

Interpersonal and Communication Skills 1: Patient- and Family-Centered Communication				
Level 1	Level 2	Level 3	Level 4	Level 5
Demonstrates respect and attempts to establish rapport	Establishes a therapeutic relationship in straightforward encounters	Establishes a culturally competent and therapeutic relationship in most encounters	Establishes a therapeutic relationship in straightforward and complex encounters, including those with ambiguity and/or conflict	Mentors others to develop positive therapeutic relationships
Attempts to adjust communication strategies based upon patient/family expectations	Adjusts communication strategies as needed to mitigate barriers and meet patient/family expectations	Communicates with sensitivity and compassion, elicits patient/family values, and acknowledges uncertainty and conflict	Uses shared decision making with patient/family to make a personalized care plan	Models and coaches others in patient- and family-centered communication

Comments:

Not Yet Completed Level 1

©2022 Accreditation Council for Graduate Medical Education (ACGME)
All rights reserved except the copyright owners grant third parties the right to use the Pediatric Endocrinology Milestones on a non-exclusive basis for educational purposes.

Interpersonal and Communication Skills 2: Patient and Family Education				
Level 1	Level 2	Level 3	Level 4	Level 5
Recognizes link between patient outcomes and education	Identifies styles and practices for effective patient education and the importance of a team approach	Educates patients using defined scripts and non-targeted methods	Tailors education to individual patients’ and their families’ needs by using varying scripts and methods	Educates patients in self-advocacy and available community resources

Comments:

Not Yet Completed Level 1

©2022 Accreditation Council for Graduate Medical Education (ACGME)
All rights reserved except the copyright owners grant third parties the right to use the Pediatric Endocrinology Milestones on a non-exclusive basis for educational purposes.

Interpersonal and Communication Skills 3: Interprofessional and Team Communication				
Level 1	Level 2	Level 3	Level 4	Level 5
Respectfully requests a consultation, with guidance	Clearly and concisely requests consultation by communicating patient information	Formulates a specific question for consultation and tailors communication strategy	Coordinates consultant recommendations to optimize patient care	Maintains a collaborative relationship with referring providers that maximizes adherence to practice recommendations
Identifies the members of the interprofessional team	Participates within the interprofessional team	Uses bi-directional communication within the interprofessional team	Facilitates interprofessional team communication	Coaches others in effective communication within the interprofessional team

Comments:

Not Yet Completed Level 1

©2022 Accreditation Council for Graduate Medical Education (ACGME)
All rights reserved except the copyright owners grant third parties the right to use the Pediatric Endocrinology Milestones on a non-exclusive basis for educational purposes.

Interpersonal and Communication Skills 4: Communication within Health Care Systems				
Level 1	Level 2	Level 3	Level 4	Level 5
Records accurate information in the patient record	Records accurate and timely information in the patient record	Concisely documents updated, prioritized, diagnostic and therapeutic reasoning in the patient record	Documents diagnostic and therapeutic reasoning, including anticipatory guidance	Models and coaches others in documenting diagnostic and therapeutic reasoning
Identifies the importance of and responds to multiple forms of communication (e.g., in-person, electronic health record (EHR), telephone, email)	Selects appropriate method of communication, with prompting	Aligns type of communication with message to be delivered (e.g., direct and indirect) based on urgency and complexity	Demonstrates exemplary written and verbal communication	Coaches others in written and verbal communication

Comments:

Not Yet Completed Level 1

©2022 Accreditation Council for Graduate Medical Education (ACGME)
All rights reserved except the copyright owners grant third parties the right to use the Pediatric Endocrinology Milestones on a non-exclusive basis for educational purposes.
