# Supplementary material for: Pediatric Endocrinology Milestones 2.0—guide to their implementation
Source: BMC Med Educ. 2023 Dec 20;23:985. doi: 10.1186/s12909-023-04862-5 (PMC10734147; doi:10.1186/s12909-023-04862-5)
Supplement: Supplementary file 2 — Additional file 2. [file 12909_2023_4862_MOESM2_ESM.pdf]

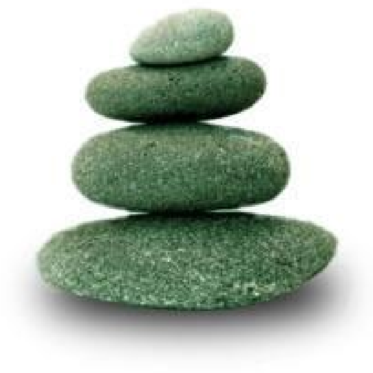

Supplemental Guide:
Pediatric Endocrinology

ACGME

April 2023

TABLE OF CONTENTS

INTRODUCTION **3**

PATIENT CARE **4**

- History 4
- Physical Exam 6
- Patient Management 7
- Diagnostic Testing 9
- Clinical Consultation 11

MEDICAL KNOWLEDGE **13**

- Physiology and Pathophysiology 13
- Clinical Reasoning 14
- Therapeutics 17

SYSTEMS-BASED PRACTICE **19**

- Patient Safety 19
- Quality Improvement 21
- System Navigation for Patient-Centered Care – Coordination of Care 23
- System Navigation for Patient-Centered Care – Transitions in Care 25
- Population and Community Health 27
- Physician Role in Health Care Systems 29

PRACTICE-BASED LEARNING AND IMPROVEMENT **31**

- Evidence-Based and Informed Practice 31
- Reflective Practice and Commitment to Personal Growth 33

PROFESSIONALISM **35**

- Professional Behavior 35
- Ethical Principles 38
- Accountability/Conscientiousness 40
- Well-Being 42

INTERPERSONAL AND COMMUNICATION SKILLS **44**

- Patient- and Family-Centered Communication 44
- Patient and Family Education 46
- Interprofessional and Team Communication 48
- Communication within Health Care Systems 51

MAPPING OF 1.0 TO 2.0 ..... **53**

RESOURCES **56**

Milestones Supplemental Guide

This document provides additional guidance and examples for the Pediatric Endocrinology Milestones. This is not designed to indicate any specific requirements for each level, but to provide insight into the thinking of the Milestone Work Group.

Included in this document is the intent of each Milestone and examples of what a Clinical Competency Committee (CCC) might expect to be observed/assessed at each level. Also included are suggested assessment models and tools for each subcompetency, references, and other useful information.

Review this guide with the CCC and faculty members. As the program develops a shared mental model of the Milestones, consider creating an individualized guide (Supplemental Guide Template available) with institution/program-specific examples, assessment tools used by the program, and curricular components.

Additional tools and references, including the Milestones Guidebook, Clinical Competency Committee Guidebook, and Milestones Guidebook for Residents and Fellows, are available at the end of this document as well as on the Resources page of the Milestones section of the ACGME website.

**Patient Care 1: History**<br>**Overall Intent:** To gather an essential and accurate patient history as it relates to a comprehensive evaluation of endocrine conditions	
**Milestones**	**Examples**
**Level 1** *Acquires a comprehensive and developmentally appropriate pediatric medical history*<br><br>*Reviews available medical records*	● In a patient referred for short stature, obtains a general medical history, family history, and comprehensive review of systems<br><br>● Reads available notes from the referring practitioner and any documentation completed by the patient pre-visit, confirming information with the patient and correcting discrepancies
**Level 2** *Acquires an endocrine history and a comprehensive pediatric medical history, including pubertal development and other pertinent positives and negatives*<br><br>*Identifies relevant findings in the medical record*	● In a female patient referred for precocious puberty, obtains timing of onset of breast development, pubic hair development or other signs, and family history of timing of growth and pubertal development<br>● Reviews growth chart to evaluate for normal versus abnormal patterns that may indicate pathology<br><br>● Extracts pertinent historical information from all primary and consultative notes, and reviews their accuracy with the patient
**Level 3** *Acquires a tailored endocrine history, including growth, historical subtleties, and psychosocial aspects*<br><br>*Independently requests additional information to supplement available medical records*	● Explores psychosocial concerns regarding peer relationships and body image in a patient with poorly controlled type 1 diabetes<br>● In a child referred for obesity, obtains neonatal/early childhood history of failure to thrive as an indicator of Prader-Willi syndrome<br><br>● Contacts the referring physician for growth charts and prior studies if not initially available
**Level 4** *Efficiently integrates the patient history with the complete medical record, supplemental information, and tailored assessment of potential endocrine disorders*	● Integrates a comprehensive history for a patient with congenital adrenal hyperplasia that addresses potential disease-, pharmacologic-, and psychosocial-related issues, and their effects on home and school life<br>● In a patient treated for prior malignancy, reviews and integrates prior treatment modalities in assessment of potential endocrinopathy
**Level 5** *Is identified as a peer resource in interpreting subtleties and recognizing ambiguities in the patient history*	● Actively participates in case discussions by providing insights into nuances of growth data as they apply to the diagnosis of growth hormone deficiency or other endocrinopathy
Assessment Models or Tools	● Direct observation<br>● Faculty member evaluations<br>● Medical record (chart) audit

	• Multisource feedback
Curriculum Mapping	•
Notes or Resources	• Textbooks<br>• American College of Rheumatology (ACR). 2020. "Patient History Form." https://www.rheumatology.org/Portals/0/Files/New%20Patient%20History%20Form.pdf. Accessed 2022.<br>• ACR. 1999. "Patient History Update." https://www.rheumatology.org/Portals/0/Files/Patient%20History%20Update%20Form.pdf. Accessed 2019.<br>• ACR. "Disease Activity and Functional Status Assessments." https://www.rheumatology.org/Practice-Quality/Clinical-Support/Quality-Measurement/Disease-Activity-Functional-Status-Assessments. Accessed 2019.<br>• ACR Ad Hoc Committee on Clinical Guidelines. 1996. "Guidelines for the Initial Evaluation of the Adult Patient with Acute Musculoskeletal Symptoms." *Arthritis and Rheumatology* 39(1): 1-8. https://doi.org/10.1002/art.1780390102.<br>• Criscione-Schreiber, Lisa. 2020. "Turning Objective Structured Clinical Examinations into Reality. *Rheumatic Diseases Clinics of North America* 46(1): 21-35. https://doi.org/10.1016/j.rdc.2019.09.010.<br>• Curran, Megan L., Kristen Hayward, and Jay Mehta. 2020. "Online Resources for Enhancing Clinical Knowledge and Skills." *Rheumatic Disease Clinics North America* 46(1): 37-60. doi: 10.1016/j.rdc.2019.09.011.<br>• Dao, Kathryn, and John J. Cush. 2006. "Acute Polyarthritis." *Best Practice and Research. Clinical Rheumatology* 20(4): 653-672. https://doi.org/10.1016/j.berh.2006.05.007.<br>• Revaz, Sylvie, Jean Dudler, and Alexander Kai-Lik So. 2006. "Fever and Musculoskeletal Symptoms in an Adult: Differential Diagnosis and Management." *Best Practice and Research. Clinical Rheumatology* 20(4): 641-651. https://doi.org/10.1016/j.berh.2006.04.006.

**Patient Care 2: Physical Examination**<br>**Overall Intent:** To perform a relevant detailed physical exam pertinent to the patient presentation	
**Milestones**	**Examples**
**Level 1** *Performs a developmentally appropriate complete physical examination, with awareness of patient comfort*	● Recognizes the need for a complete physical examination in a child referred for short stature<br>● Identifies the need for examination of puberty staging in the presence of a chaperone
**Level 2** *Performs a developmentally appropriate complete physical examination using strategies to optimize patient comfort and identifies abnormal endocrine findings*	● Discusses the need for assessment of pubertal development with the patient and patient's family prior to examination<br>● Identifies breast buds in a six-year-old girl as a sign of precocious puberty
**Level 3** *Performs a tailored physical examination using strategies to optimize patient comfort and identifies subtle abnormal endocrine findings*	● Examines pump or injection sites in a patient with type 1 diabetes and identifies lipohypertrophy or lipoatrophy<br>● Routinely performs thyroid examinations in patients with Turner syndrome<br>● Recognizes that a detailed pubertal examination is not required for all patients at every visit
**Level 4** *Detects, pursues, and integrates key physical examination findings to distinguish nuances among competing, often similar diagnoses*	● Assesses hyperpigmentation in a patient with potential adrenal insufficiency, understanding that this may present differently in patients of color<br>● Evaluates for micropenis in a patient with neonatal hypoglycemia to assess for congenital hypopituitarism
**Level 5** *Is identified as a peer resource for performing tailored physical exams, maximizing patient comfort*	● Is identified by the program director to lead a medical student thyroid exam workshop
Assessment Models or Tools	● Direct observation<br>● Faculty member evaluations<br>● Medical record (chart) audit
Curriculum Mapping	●
Notes or Resources	● Textbooks<br>● Online resources<br>● Workshops<br>● Standardized outcome measures (e.g., tender and swollen joint counts, modified Rodnan skin score)<br>● Villaseñor-Ovies, Pablo, José Eduardo Navarro-Zarza, and Juan J. Canoso. 2019. "The Rheumatology Physical Examination: Making Clinical Anatomy Relevant." *Clinical Rheumatology* 39(3): 651–657. https://doi.org/10.1007/s10067-019-04725-9.

Patient Care 3: Patient Management<br>**Overall Intent:** To lead the health care team in the creation of a comprehensive, patient-centered management plan based on multiple patient factors, including social factors and varied patient backgrounds, regardless of complexity	
**Milestones**	**Examples**
**Level 1** *Reports and implements management plans developed by others for routine endocrine presentations*	• Asks for supervisor's management plan without proposing own plans or suggestions and relays to patient and family
**Level 2** *Develops and implements management plans that require modification for routine endocrine presentations*	• For a patient with new onset diabetes, proposes doses of long-acting and rapid-acting insulin that require some adjustment by attending<br>• Recommends standing orders for desmopressin (DDAVP) administration without considering possible variations in urine output
**Level 3** *Develops and implements management plans for routine endocrine presentations*	• For a patient with new diabetes, appropriately prescribes long-acting and rapid-acting insulin and conveys lab recommendations to inpatient team<br>• For a patient with hypothyroidism, orders an appropriate dose of levothyroxine and follow-up labs
**Level 4** *Develops and implements management plans for complex endocrine presentations, and modifies plans as necessary*	• For a post-operative neurological surgery patient, creates initial plans for management of fluid and sodium and adjusts as needed based on clinical course; recommends vasopressin when the patient develops polyuria and hypernatremia, without need for additional input from the supervising attending<br>• For a patient with hypothyroidism secondary to thyroidectomy due to thyroid cancer, recognizes the need for and orders a higher dose of levothyroxine
**Level 5** *Is identified as a peer resource for development of management plans for complex endocrine presentations, and modifies plans as necessary*	• Is recognized by the faculty members as an expert in providing appropriate advice to other fellows, and in helping them think through the management plan for a complicated consult<br>• During case conference, correctly recommends evidence-based management when a complicated inpatient is presented
Assessment Models or Tools	• Case-based discussions<br>• Direct observation<br>• End-of-rotation evaluations<br>• Medical record (chart) audit
Curriculum Mapping	•
Notes or Resources	• American Board of Pediatrics. "Entrustable Professional Activities for Subspecialties." Pediatric Endocrinology, EPA 3 and EPA 4. https://www.abp.org/content/entrustable-professional-activities-subspecialties. Accessed 2021.

	• Cook, David A., Steven J. Durning, Johnathan Sherbino, and Larry D. Gruppen. 2019. "Management Reasoning: Implications for Health Professions Educators and a Research Agenda." *Academic Medicine* 94(9): 1310–1316. doi: 10.1097/ACM.0000000000002768.

**Patient Care 4: Diagnostic Testing, Including Labs, Imaging, and Functional Testing**<br>**Overall Intent:** To perform and interpret appropriate laboratory, radiology, and functional testing to inform the differential diagnosis	
**Milestones**	**Examples**
**Level 1** *Orders non-targeted tests for patients with routine endocrine presentations*	● In a patient with routine hypothyroidism follow-up, suggests ordering thyroid-stimulating hormone (TSH), free T4, total T3, and thyroid antibodies<br>● In a girl with isolated premature adrenarche, orders androgens, luteinizing hormone (LH), follicle-stimulating hormone (FSH), and estradiol
Interprets basic endocrine test results, with guidance	● In a patient with a TSH of 20uU/mL, knows this indicates hypothyroidism but not sure of etiology or severity
**Level 2** *Orders targeted tests for patients with routine endocrine presentations*	● Orders only free thyroxine level to monitor replacement for a patient with central hypothyroidism<br>● Orders diabetes antibodies and C-peptide/glucose test to identify etiology of diabetes in an adolescent with obesity
Independently interprets targeted test results for routine endocrine presentations	● For a patient with central hypothyroidism with a free T4 in the lower normal range and a low TSH, suggests a small increase in levothyroxine
**Level 3** *Orders targeted tests for patients with complex endocrine presentations*	● In a patient with concern for Cushing disease, obtains appropriate initial testing based on guidelines<br>● In a patient receiving cranial radiation for suprasellar tumor, orders appropriate labs to assess pituitary function
Interprets targeted test results for patients with complex endocrine presentations, with assistance, and identifies incongruencies	● In an infant with ambiguous genitalia and no palpable gonads, knows that a 17-hydroxyprogesterone (OHP) of 20,000 ng/dL indicates classical congenital adrenal hyperplasia (CAH)<br>● In a patient with obesity and normal growth, recognizes that a mildly elevated 24-hour urine free cortisol is not indicative of Cushing disease
**Level 4** *Develops individualized cost-effective testing strategies to evaluate patients with complex endocrine presentations and avoids unnecessary testing*	● In a patient with known autoimmune hypothyroidism and a goiter, does not routinely obtain a thyroid ultrasound
Resolves incongruencies and accepts ambiguity in targeted test results for patients with complex endocrine presentations	● Recognizes when thyroid function tests do not align with the clinical presentation and considers biotin or other assay interference<br>● Brings conflicting results on a patient to case conference to discuss differential diagnosis and appropriate management

**Level 5** *Identifies, critically evaluates, and selectively uses emerging and investigational tests or procedures; questions and reports unknown and unexplained discrepancies*	• Investigates use of copeptin as a tool for diagnosis in a patient with diabetes insipidus<br>• Uses molecular testing to determine surgical plan for indeterminant thyroid nodules
Assessment Models or Tools	• Case-based discussions<br>• Direct observation<br>• End-of-rotation evaluations<br>• Medical record (chart) audit
Curriculum Mapping	•
Notes or Resources	• American Board of Pediatrics. "Entrustable Professional Activities for Subspecialties." Pediatric Endocrinology, EPA 1. https://www.abp.org/content/entrustable-professional-activities-subspecialties. Accessed 2021.<br>• Ergin, Ahmet Bahadir, A. Laurence Kennedy, Manjula K. Gupta, and Amir H. Hamrahian. 2015. *The Cleveland Clinic Manual of Dynamic Endocrine Testing,* 2015 ed. Switzerland: Springer.<br>• Sluss, P.M., and F.J. Hayes. 2019. "Laboratory Techniques for Recognition of Endocrine Disorders." In: Melmed, S., R. Koenig, C. Rosen, R. Auchus, and A. Goldfine. *Williams Textbook of Endocrinology*. 14th ed. Elsevier.<br>• Soh, Shui Boon, and Tar Choon Aw. 2019. "Laboratory Testing in Thyroid Conditions - Pitfalls and Clinical Utility." *Annals of Laboratory Medicine* 39(1): 3-14. https://doi.org/10.3343/alm.2019.39.1.3. PMID: 30215224; PMCID: PMC6143469.<br>• Yeo, Kiang-Tech J., Nikolina Babic, Zeina C. Hannoush, and Roy E. Weiss. Updated 2017. *Endocrine Testing Protocols: Hypothalamic Pituitary Adrenal Axis*. South Dartmouth, MA: MDText.com, Inc. https://www.ncbi.nlm.nih.gov/books/NBK278940/.

Patient Care 5: Clinical Consultation<br>**Overall Intent:** To provide comprehensive consultative care for patients in the inpatient and outpatient settings	
**Milestones**	**Examples**
**Level 1** *Responds to consultation after receiving assistance*	● Responds to requests in a timely and courteous manner<br>● Requires guidance on all aspects of consultation including gathering pertinent information, appropriate evaluation, management, and communication with the patient/family and requesting practitioner
Recognizes disease acuity, with supervision	● Confirms with attending that an outpatient referral for hyperthyroidism should be seen promptly
**Level 2** *Clarifies the clinical questions and provides preliminary recommendations to the requesting practitioner*	● Politely asks clarifying questions during a consult request call from an inpatient service and provides the team with initial laboratory evaluation without prior discussion with the attending physician, then proceeds to discuss the case with the attending physician and complete the consultation
Independently recognizes disease acuity	● Independently returns to hospital after hours to evaluate a patient with suspected thyroid storm<br>● Recognizes that a seven-year-old female with isolated breast budding does not require immediate evaluation
**Level 3** *Seeks and integrates input from different members of the health care team and provides recommendations to the requesting practitioner in a clear and timely manner*	● For a patient with hyperthyroidism, not at treatment goal, and thyroid eye disease, refers to ophthalmology for consultation and integrates input to develop a treatment plan<br>● Discusses options with a multidisciplinary team, including urology, genetics, gynecology, and psychology, for a patient with a difference of sex development
Recognizes disease acuity and prioritizes management steps	● For a patient with suspected thyroid storm, presents case to the attending and recommends patient be admitted to the intensive care unit (ICU)<br>● For a seven-year-old female with isolated breast budding, assists a primary care practitioner in developing a follow-up plan in the primary care setting
**Level 4** *Provides comprehensive and prioritized recommendations, including assessment, rationale, and anticipatory guidance to all relevant health care team members*	● Monitors patient progress closely and modifies treatment plan as indicated, including heart rate and blood pressure monitoring with beta blocker in the treatment of hyperthyroidism<br>● Ensures patient's family and health care team members receive anticipatory guidance for use of stress dose steroids
Mobilizes resources based on acuity of the situation	● Considers costs of studies and therapies for patients with limited means and identifies community resources

	• Discusses need for monitoring and prompt treatment for a patient with severe diabetic ketoacidosis and ensures appropriate monitoring for neurologic status<br>• Recommends transfer of an infant with severe hypocalcemia to a tertiary care center
**Level 5** *Is identified as a peer resource for the provision of consultative care across the spectrum of disease complexity and acuity*	• Provides education to team members regarding the diagnosis and management of hyperthyroidism
Assessment Models or Tools	• Direct observation<br>• Evaluation of case-based discussion or conference presentation<br>• End-of-rotation evaluation<br>• Medical record (chart) audit<br>• Multisource feedback
Curriculum Mapping	•
Notes or Resources	• Endocrine Society. "Clinical practice guidelines." https://www.endocrine.org/clinical-practice-guidelines. Accessed 2020.<br>• Sluss, P.M., and F.J. Hayes. 2019. "Laboratory Techniques for Recognition of Endocrine Disorders." In: Melmed, S., R. Koenig, C. Rosen, R. Auchus, and A. Goldfine. *Williams Textbook of Endocrinology*. 14th ed. Elsevier.

Medical Knowledge 1: Physiology and Pathophysiology<br>**Overall Intent:** To demonstrate knowledge of physiology and pathophysiology through integration with diagnosis and management	
**Milestones**	**Examples**
**Level 1** *Demonstrates generalized knowledge of physiological and pathophysiological concepts in endocrinology*	● Describes basics of hormonal feedback loop
**Level 2** *Demonstrates knowledge of physiology and pathophysiology of routine endocrine conditions*	● Explains normal adrenal function and identifies changes in adrenal function that occur in patients with adrenal insufficiency<br>● Enumerates the hormonal regulation of calcium metabolism in a patient with hypocalcemia
**Level 3** *Applies knowledge of physiology and pathophysiology to diagnosis and management of routine presentations*	● Understands regulation of glucose metabolism and mechanism of action of insulin to develop an appropriate insulin regimen for a patient with type 1 diabetes
**Level 4** *Applies knowledge of physiology and pathophysiology to diagnosis and management of complex presentations*	● Synthesizes the pathophysiology of insulin action and uses this knowledge to understand the influence of obesity on management of type 1 diabetes; considers additional pharmacologic and non-pharmacologic treatments<br>● In a patient with hypoglycemia undergoing diagnostic fast, explains how an elevation of blood glucose to glucagon leads to a diagnosis of hyperinsulinism
**Level 5** *Synthesizes newly described and emerging clinical physiology and pathophysiology concepts with diagnosis and management*	● Identifies clinical trials or experimental treatments for patients with rare endocrine diseases based on scientific evidence<br>● Collaborates with or participates in multicenter trials
Assessment Models or Tools	● Board review<br>● Case presentations<br>● Direct observation<br>● In-training exam<br>● Medical record (chart) audit
Curriculum Mapping	
Notes or Resources	● Pediatric Endocrine Society. “Clinical Resource Library.” https://pedsendo.org/clinical-resources/. Accessed 2022.<br>● Sperling, Mark A. 2020. *Sperling Pediatric Endocrinology*. 5th ed. Elsevier. https://doi.org/10.1016/C2017-0-02772-6 .

**Medical Knowledge 2: Clinical Reasoning**<br>**Overall Intent:** To consistently develop a complete and prioritized differential diagnosis while minimizing the impact of cognitive errors	
**Milestones**	**Examples**
**Level 1** *Organizes and accurately summarizes information obtained from the patient evaluation to develop a clinical impression*	● After evaluating a patient for abnormal newborn screen with elevated 17-OHP and atypical genitalia, develops the clinical impression of congenital adrenal hyperplasia
**Level 2** *Integrates information from all sources to develop a basic differential diagnosis for routine endocrine presentations*<br><br>*Identifies clinical reasoning errors within patient care, with guidance*	● Uses patient history, growth charts from the primary care physician, and physical exam findings to develop a differential diagnosis for nine-year-old girl with poor growth<br><br>● In discussion with senior physician, identifies own lack of awareness as reason for not including Turner syndrome in the differential diagnosis of a nine-year-old girl with poor growth<br>● In discussion with clinic attending, recognizes own implicit bias as a reason for not considering pathologic causes of short stature in a Latina girl presenting with poor growth
**Level 3** *Develops a thorough and prioritized differential diagnosis for routine endocrine presentations*<br><br>*Retrospectively applies clinical reasoning principles to identify errors*	● For a nine-year-old girl with history of horseshoe kidney presenting to clinic for evaluation of poor growth, discusses the differential of short stature and identifies Turner syndrome as one of the high probability causes of short stature in this patient<br><br>● During a team discussion of a patient with type 1 diabetes, recognizes that accurate diagnosis was delayed due to anchoring on a presumptive diagnosis of type 2 diabetes in an adolescent with obesity<br>● After expression of frustration with a patient for “non-compliance” with diet and exercise recommendations, asks patient about access to food and safe and accessible areas for exercise
**Level 4** *Synthesizes subtle, unusual, or conflicting findings to prioritize differential diagnoses in complex endocrine presentations*<br><br>*Continually re-appraises own clinical reasoning to improve patient care in real time*	● Identifies hypopituitarism as a cause of presentation for a six-month-old boy presenting for failure to thrive and history of seizures found to have hyperbilirubinemia and micropenis<br><br>● After a recent missed diagnosis of pseudohypoparathyroidism, considers this diagnosis for a patient presenting with obesity and TSH abnormalities

	• For a seven-year-old boy followed for growth hormone deficiency, recognizes that persistent hyponatremia can signal other pituitary deficiencies and require further evaluation<br>• When a patient's weight percentile continues to rise despite appropriate lifestyle counseling, asks patient and patient's family about access to food and safe and accessible areas for exercise
**Level 5** *Coaches others to develop prioritized differential diagnoses in complex endocrine presentations*<br><br>*Models how to recognize errors and reflect upon one's own clinical reasoning*	• Teaches a PGY-1 resident to link associations in history, physical exam, and biochemical testing in order to hone differential diagnosis in a patient with dysnatremia<br><br>• For a nine-year-old boy with adrenal insufficiency, articulates that the diagnosis of X-linked adrenoleukodystrophy was delayed due to anchoring on the diagnosis of autoimmune adrenal insufficiency due to a history of Hashimoto's thyroiditis, and discusses how to change the evaluation to include other potential diagnoses
Assessment Models or Tools	• Chart-stimulated recall<br>• Direct observation<br>• Evaluation of formal case presentations incorporating explicit discussion of clinical reasoning (case conferences, morbidity and mortality (M and M) conferences, etc.)<br>• Medical record (chart) audit<br>• Multisource feedback<br>• Reflection
Curriculum Mapping	•
Notes or Resources	• American College of Physicians (ACP). "Getting it Right: Cases to Improve Diagnosis." https://www.acponline.org/cme-moc/online-learning-center/getting-it-right-cases-to-improve-diagnosis. Accessed 2020.<br>• ACP. "Teaching Clinical Reasoning." https://store.acponline.org/ebiz/products-services/product-details/productid/21910?productId=21910. Accessed 2020.<br>• Bowen, Judith. 2006. "Educational Strategies to Promote Clinical Diagnostic Reasoning." *New England Journal of Medicine* 355(21): 2217-2225. doi:10.1056/NEJMra054782.<br>• Charlin, B., J. Tardif, and H.P. Boshuizen. 2000. "Scripts and Medical Diagnostic Knowledge: Theory and Applications for Clinical Reasoning Instruction and Research." *Academic Medicine* 75(2): 182-190. https://doi.org/10.1097/00001888-200002000-00020.<br>• Croskerry, Pat. 2009. "A Universal Model of Diagnostic Reasoning." *Academic Medicine* 84(8):1022-1028. https://doi.org/10.1097/ACM.0b013e3181ace703.<br>• DocNomo phone app

	● Graber, Mark L., Nancy Franklin, and Ruthanna Gordon. 2005. “Diagnostic Error in Internal Medicine.” *Archives of Internal Medicine* 165(13):1493-1499. doi:10.1001/archinte.165.13.1493.<br>● Mamede, Silvia, Henk G. Schmidt, and Júlio César Penaforte. 2008. “Effects of Reflective Practice on the Accuracy of Medical Diagnosis.” *Medical Education* 42(5): 468-475. https://doi.org/10.1111/j.1365-2923.2008.03030.x.<br>● Norman, Geoffrey, Sandra Monteiro, Jonathan Sherbino, Jonathan Seth Ilgen, Henk G. Schmidt, and Silvia Mamede. 2017. “The Causes of Errors in Clinical Reasoning: Cognitive Biases, Knowledge Deficits, and Dual Process Thinking.” *Academic Medicine* 92(1):23-30. doi:10.1097/ACM.0000000000001421.<br>● Society to Improve Diagnosis in Medicine. https://www.improvediagnosis.org/. Accessed 2020.

**Medical Knowledge 3: Therapeutics (Behavioral, Medications, Technology, Radiopharmaceuticals)**<br>**Overall Intent:** To appropriately prescribe and manage therapeutics for endocrine conditions	
**Milestones**	**Examples**
**Level 1** *Demonstrates knowledge of basic endocrine therapeutics*	● Is aware that levothyroxine treatment is a daily oral medication<br>● Understands basic utilization of insulin in the treatment of diabetes<br>● Understands how culture and/or religious belief may impact a patient's diet when managing obesity<br>● Understands that where a patient lives (urban versus rural) influences recommendations regarding physical activity
**Level 2** *Demonstrates knowledge of the indications, monitoring parameters, and adverse effects of endocrine therapeutics*	● Is aware that thyroid labs should be obtained to assess levothyroxine dose and is aware of the adverse effects of overtreatment<br>● Is aware that growth hormone treatment is not needed in a patient with constitutional delay of growth and puberty
**Level 3** *Applies knowledge of therapeutics to the management of patients with routine endocrine conditions*	● Prescribes methimazole and atenolol for the initial treatment of Graves' disease in a patient with history of asthma<br>● Initiates correct therapy for precocious puberty, monitoring with serial heights, physical examination, and when indicated, biochemical parameters
**Level 4** *Applies knowledge of therapeutics to the management of patients with complex endocrine conditions*	● Assesses for adrenal insufficiency prior to starting therapy with levothyroxine in a patient with hypopituitarism<br>● Adjusts insulin dosage in a patient with diabetes and kidney failure<br>● Monitors prolactin levels and reviews visual field testing and magnetic resonance imaging (MRI) results when following a patient treated with cabergoline for prolactinoma
**Level 5** *Identifies targeted or experimental therapies for complex and rare clinical scenarios*	● Recommends treatment with asfotase alfa for an infant with hypophosphatasia
Assessment Models or Tools	● Board review<br>● Direct observation<br>● End-of-rotation evaluations<br>● Evaluation of conference presentations<br>● In-training exam<br>● Medical record (chart) audit<br>● Multisource feedback
Curriculum Mapping	●
Notes or Resources	● American Association of Clinical Endocrinology (AACE). "Disease State Resource Centers." https://pro.aace.com/resources. Accessed 2020.<br>● Endocrine Society. "Clinical Practice Guidelines." https://www.endocrine.org/clinical-practice-guidelines. Accessed 2020.

	● Ospina, N.S., S. Maraka, R. Rodriguez-Gutierrez, J.P. Brito, and V. Montori. 2019. “Navigating Through Clinical Practice Guidelines in Endocrinology.” In: Melmed, S., R. Koenig, C. Rosen, R. Auchus, and A. Goldfine. *Williams Textbook of Endocrinology*. 14th ed. Elsevier.<br>● Whittier, D.E., S.K. Boyd, A.J. Burghardt, J. Paccou, A. Ghasem-Zadeh, R. Chapurlat, K. Engelke, and M.L. Bouxsein. 2020. “Guidelines for the Assessment of Bone Density and Microarchitecture in Vivo Using High-Resolution Peripheral Quantitative Computed Tomography.” *Osteoporosis International* 31(9):1607-1627. https://doi.org/10.1007/s00198-020-05438-5.

**Systems-Based Practice 1: Patient Safety**<br>**Overall Intent:** To engage in the analysis and management of patient safety events, including relevant communication with patients, patients' families, and health care professionals	
**Milestones**	**Examples**
**Level 1** *Demonstrates knowledge of common patient safety events*<br><br>*Demonstrates knowledge of how to report patient safety events*	● Identifies medication errors as major safety events in diabetes mellitus and diabetes insipidus<br><br>● Lists "patient safety reporting system" or "patient safety hotline" as ways to report safety events
**Level 2** *Identifies system factors that lead to patient safety events*<br><br>*Reports patient safety events through institutional reporting systems (simulated or actual)*	● Identifies that electronic health record (EHR) default timing of orders may lead to delays in insulin administration time<br>● Identifies limitations in ability to obtain a critical sample in a patient with hypoglycemia<br><br>● Reports delayed insulin administration time using the appropriate reporting mechanism
**Level 3** *Participates in analysis of patient safety events (simulated or actual)*<br><br>*Participates in disclosure of patient safety events to patients and families (simulated or actual)*	● Participates in root cause analyses (mock or actual) of incorrect medication administration<br>● Participates in a quality improvement project aimed at reducing racial disparities<br><br>● With the support of an attending or risk management team member, participates in the disclosure of a medication order error to a patient's family
**Level 4** *Conducts analysis of patient safety events and offers error prevention strategies (simulated or actual)*<br><br>*Discloses patient safety events to patients and families (simulated or actual)*	● Leads a simulated or actual root cause analysis related to a patient who receives an inappropriate insulin dose and suffers a hypoglycemic seizure, and develops an action plan that includes ensuring appropriate input of insulin orders in the EHR, nursing verification, and patient identification<br><br>● Following consultation with risk management and other team members, independently discloses a medication error to a patient's family
**Level 5** *Actively engages teams and processes to modify systems to prevent patient safety events*<br><br>*Role models or mentors others in the disclosure of patient safety events*	● Leads a multidisciplinary team to develop system-wide action plans for adrenal insufficiency and stress dosing<br><br>● Conducts a simulation demonstrating techniques and approaches for disclosing patient safety events
Assessment Models or Tools	● Case-based discussions

	• Direct observation<br>• E-module multiple choice tests<br>• Guided reflection<br>• Medical record (chart) audit<br>• Multisource feedback<br>• Simulation
Curriculum Mapping	•
Notes or Resources	• American Board of Pediatrics. "Entrustable Professional Activities for Subspecialties." Pediatric Endocrinology. https://www.abp.org/content/entrustable-professional-activities-subspecialties. Accessed 2021.<br>• Institute of Healthcare Improvement. http://www.ihi.org/Pages/default.aspx. Accessed 2020.<br>• Singh, Ranjit, Bruce Naughton, John S. Taylor, Marlon R. Koenigsberg, Diana R. Anderson, Linda L. McCausland, Robert G. Wahler, Amanda Robinson, and Gurdev Singh. 2005. "A Comprehensive Collaborative Patient Safety Residency Curriculum to Address the ACGME Core Competencies." *Medical Education* 39(12): 1195-204. https://doi.org/10.1111/j.1365-2929.2005.02333.x.

**Systems-Based Practice 2: Quality Improvement**<br>**Overall Intent:** To understand and implement quality improvement methodologies to improve patient care	
**Milestones**	**Examples**
**Level 1** *Demonstrates knowledge of basic quality improvement methodologies and metrics*	● Describes fishbone diagram<br>● Describes components of a “Plan-Do-Study-Act” cycle
**Level 2** *Describes local quality improvement initiatives (e.g., insulin management, screening for diabetes complications)*	● Describes clinic initiatives to improve flu vaccination rates for patients with type 1 diabetes<br>● Describes an initiative to improve collection of urine samples for microalbumin in adolescents with type 2 diabetes
**Level 3** *Participates in local quality improvement initiatives*	● Participates in a quality improvement project to review TSH data in patients with congenital hypothyroidism<br>● Collaborates on a project to improve stress dose teaching at discharge for patients with adrenal insufficiency
**Level 4** *Demonstrates the skills required to identify, develop, implement, and analyze a quality improvement project*	● Develops and implements a quality improvement project to improve TSH screening in neonates exposed to iodine, including engaging the neonatal intensive care unit (NICU) staff and cardiology staff; assessing the problem; articulating a broad goal; developing a SMART (Specific, Measurable, Attainable, Realistic, Time-bound) aim; collecting data; and analyzing and monitoring progress and challenges<br>● In developing a quality improvement project, considers team bias and social determinants of health in patient population
**Level 5** *Creates, implements, and assesses quality improvement initiatives at the institutional or community level*	● Initiates and completes a quality improvement project to improve diabetes knowledge among school nurses, and shares results through a formal presentation to the hospital and community leaders<br>● Looks for opportunities to improve flu vaccination rates across a health care system<br>● Consistently engages in quality improvement around improving flu vaccination rates
Assessment Models or Tools	● Direct observation<br>● E-module multiple choice test<br>● Portfolio<br>● Poster or other presentation<br>● Team evaluations
Curriculum Mapping	●
Notes or Resources	● American Academy of Pediatrics. “Bright Futures.” https://www.aap.org/en/practice-management/bright-futures. Accessed 2020.<br>● American Board of Pediatrics. “Entrustable Professional Activities for Subspecialties.” Pediatric Endocrinology. https://www.abp.org/content/entrustable-professional-activities-subspecialties. Accessed 2021.

	• Institute of Healthcare Improvement. http://www.ihi.org/Pages/default.aspx. Accessed 2020.<br>• Murtagh Kurowski, Eileen, Amanda C. Schondelmeyer, Courtney Brown, Christopher E. Dandoy, Samuel J. Hanke, and Heather L. Tubbs Cooley. 2015. "A Practical Guide to Conducting Quality Improvement in the Health Care Setting." *Current Treatment Options in Pediatrics* 1:380-392. https://doi.org/10.1007/s40746-015-0027-3.

**Systems-Based Practice 3: System Navigation for Patient-Centered Care – Coordination of Care**<br>**Overall Intent:** To effectively navigate the health care system, including the interdisciplinary team and other care practitioners; to adapt care to a specific patient population to ensure high-quality patient outcomes	
**Milestones**	**Examples**
**Level 1** *Lists the various interprofessional individuals involved in the patient's care coordination*	● For a patient with diabetes, identifies the team members and roles as part of the team, including diabetes educator, mental health practitioner, nutritionist, nurses, and pediatric endocrinologist<br>● Identifies important members of the medical home team for a patient with complex conditions in the continuity clinic<br>● Recognizes implicit bias as a contributor to health care disparities<br>● Identifies access to care and insurance coverage as social determinants of health
**Level 2** *Coordinates care of patients in routine clinical situations, incorporating interprofessional teams with consideration of patient and family needs*	● After a new diagnosis of type 1 diabetes, coordinates care with the inpatient team and the outpatient clinic team<br>● For a family with multiple stressors after a diagnosis of type 1 diabetes, adds a social worker and mental health practitioner to the care team
**Level 3** *Coordinates care of patients in complex clinical situations, effectively utilizing the roles of interprofessional teams, and incorporating patient and family needs and goals*	● Works with the social worker to coordinate outpatient care and ensure appropriate endocrine clinic follow-up for a patient with panhypopituitarism who resides in a rural area with limited family transportation options<br>● Recognizes that patients from underserved communities may have additional barriers to access and the need to involve a social worker or case manager in finding community resources
**Level 4** *Coordinates interprofessional, patient-centered care among different disciplines and specialties, actively assisting families in navigating the health-care system*	● Recognizes the need for and coordinates a multidisciplinary team/family meeting to include appropriate subspecialists, physical therapist/occupational therapist, nutrition, child life, mental health resources, chaplain services, the primary care physician, etc.<br>● Refers patients to a local pharmacy that offers a sliding fee scale and provides pharmacy coupons for patients in need<br>● Writes a letter of necessity to the insurance company and participates in a peer-to-peer review of request for treatment
**Level 5** *Coaches others in interprofessional, patient-centered care coordination*	● Leads outreach to school nurses educating children about diabetes care<br>● Coaches and mentors colleagues through a multidisciplinary team meeting about a child with a difference of sex development
Assessment Models or Tools	● Direct observation<br>● Multisource feedback<br>● Review of discharge planning documentation
Curriculum Mapping	●
Notes or Resources	● American Academy of Pediatrics (AAP). https://www.aap.org/en-us/Pages/Default.aspx. Accessed 2020.

	● American Board of Pediatrics. "Entrustable Professional Activities for Subspecialties." Pediatric Endocrinology. https://www.abp.org/content/entrustable-professional-activities-subspecialties. Accessed 2021.<br>● Skochelak, Susan E., Maya M. Hammond, Kimberly D. Lomis, Jeffrey M. Borkan, Jed. D. Gonzalo, Luan E. Lawson, and Stephanie R. Starr. 2020. *AMA Education Consortium: Health Systems Science*, 2nd ed. Elsevier.<br>● Starr, Stephanie R., Neera Agrwal, Michael J. Bryan, Yuna Buhrman, Jack Gilbert, Jill M. Huber, and Andrea N. Leep Hunderfund. 2017. "Science of Health Care Delivery: An Innovation in Undergraduate Medical Education to Meet Society's Needs." *Mayo Clinic Proceedings: Innovations, Quality & Outcomes* 1(2): 117-129. https://doi.org/10.1016/j.mayocpiqo.2017.07.001.

**Systems-Based Practice 4: System Navigation for Patient-Centered Care – Transitions in Care**<br>**Overall Intent:** To effectively navigate the health delivery system during transitions of care to ensure high-quality patient outcomes	
**Milestones**	**Examples**
**Level 1** *Uses a standard template for transitions of care/hand-offs*	• When handing off to colleagues for the weekend call, reads verbatim from a templated hand-off but lacks contingency plans
**Level 2** *Adapts a standard template, recognizing key elements for safe and effective transitions of care/hand-offs in routine clinical situations*	• Routinely uses a standardized hand-off for a stable patient, verbalizes an understanding of active problems, and provides basic contingency plans<br>• Discusses the discharge of a patient with diabetes from the ward with the primary pediatric endocrinologist and provides the clinical course and action items to be followed up as an outpatient
**Level 3** *Performs safe and effective transitions of care/hand-offs in complex clinical situations, and ensures closed-loop communication*	• When handing off a post-surgical patient who is newly diagnosed with craniopharyngioma, routinely uses direct communication about clinical reasoning, problems warranting a higher level of care, and status of completed/planned interventions; solicits read-back and confirms/uses specific resources and timeline for transfer to occur
**Level 4** *Performs and advocates for safe and effective transitions of care/hand-offs within and across health care delivery systems, including transitions to adult care*	• Prior to going on vacation, proactively seeks out colleagues to follow up on test results expected back during that week with specific instructions and contingency plans for the follow-up visit with the patient/family<br>• Seeks out appropriate adult endocrinology practitioners to facilitate the transition of a 22-year-old patient with type 1 diabetes to adult care; ensures a thorough hand-off, including the patient's cultural preferences and social needs, to the identified new adult practitioners
**Level 5** *Coaches others in improving transitions of care within and across health care delivery systems to optimize patient outcomes*	• Designs and implements standardized hand-off workshop exercises for learners prior to the start of their clinical rotations<br>• Develops and implements a process to improve the transition from pediatric to adult endocrinology
Assessment Models or Tools	• Direct observation<br>• Portfolio assessment<br>• Multisource feedback<br>• Review of sign-out tools, use and review of checklists<br>• Standardized hand-off checklist
Curriculum Mapping	•
Notes or Resources	• American Board of Pediatrics. "Entrustable Professional Activities for Subspecialties." Pediatric Endocrinology. https://www.abp.org/content/entrustable-professional-activities-subspecialties. Accessed 2021.<br>• GotTransition. "Clinician Education & Resources." https://www.gottransition.org/resources-and-research/clinician-education-resources.cfm. Accessed 2020.

	• Matern, Lukas H., Jeanne M. Farnan, Kristen W. Hirsch, Melissa Cappaert, Ellen S. Byrne, and Vineet M. Arora. 2018. "A Standardized Handoff Simulation Promotes Recovery from Auditory Distractions in Resident Physicians." *Simulation in Healthc*are 13(4): 233-238. doi: 10.1097/SIH.0000000000000322.<br>• Society for Adolescent Health and Medicine. 2020. "Transition to Adulthood for Youth with Chronic Conditions and Special Health Care Needs." *Journal of Adolescent Health*. 66(5): P631-634. https://doi.org/10.1016/j.jadohealth.2020.02.006.<br>• Starmer, Amy J., Nancy D. Spector, Rajendu Srivastava, Daniel C. West, Glenn Rosenbluth, April D. Allen, Elizabeth L. Noble, et al. "Changes in Medical Errors after Implementation of a Handoff Program." *New England Journal of Med*icine 371: 1803-1812. doi: 10.1056/NEJMsa1405556.

Systems-Based Practice 5: Population and Community Health<br>**Overall Intent:** To promote and improve health across communities and populations through patient care and advocacy, including public education and elimination of structural racism	
**Milestones**	**Examples**
**Level 1** *Demonstrates awareness of population and community health needs and disparities*	• Recognizes poverty and structural racism as examples of social determinants of health<br>• Recognizes parental divorce and housing insecurity as examples of adverse childhood experiences
**Level 2** *Identifies specific population and community health needs and disparities; identifies local resources*	• Recognizes social and economic contributors to obesity in underserved and disadvantaged populations<br>• Determines patient is having food insecurity and refers the patient to social workers
**Level 3** *Uses local resources effectively to meet the needs and reduce health disparities of a patient population and community*	• Refers an uninsured patient with new type 1 diabetes to a patient assistance program and/or organizations that can provide free or lower-cost insulin<br>• Knows alternative lower-cost insulin regimens for a patient on an insulin pump who has lost insurance coverage
**Level 4** *Adapts practice to provide for the needs of and reduce health disparities of a specific population*	• Participates in an advocacy project to improve health care access and/or decrease practices that support structural racism<br>• Identifies available mental health resources for patients who screen positive for depression on Patient Health Questionnaire-9 (PHQ-9)
**Level 5** *Advocates at the local, regional, or national level for populations and communities with health care disparities*	• Engages in a project to open a food pantry<br>• Partners with a community organization working to increase flu vaccination rates for a particular group<br>• Participates in longitudinal discussions with local, state, or national government policy makers to eliminate structural racism and reduce health disparities
Assessment Models or Tools	• Case conference discussions<br>• Direct observation<br>• Multisource feedback<br>• Participation in advocacy<br>• Reflection
Curriculum Mapping	•
Notes or Resources	• AAP. "Bright Futures." https://www.aap.org/en/practice-management/bright-futures. Accessed 2020.<br>• AAP. "Advocacy." https://services.aap.org/en/advocacy/. Accessed 2020.<br>• American Board of Pediatrics. "Entrustable Professional Activities for Subspecialties." Pediatric Endocrinology. https://www.abp.org/content/entrustable-professional-activities-subspecialties. Accessed 2021.<br>• Blankenburg, Rebecca, Patricia Poitevien, Javier Gonzalez del Rey, Megan Aylor, John Frohna, Heather McPhillips, Linda Waggoner-Fountain, and Laura Degnon. 2020.

	"Dismantling Racism: Association of Pediatric Program Directors' Commitment to Action." *Academic Pediatrics.* 20(8): 1051-1053. doi: 10.1016/j.acap.2020.08.017.<br>● Centers for Disease Control and Prevention. "Preventing Adverse Childhood Experiences." https://www.cdc.gov/violenceprevention/aces/fastfact.html?CDC_AA_refVal=https%3A%2F%2Fwww.cdc.gov%2Fviolenceprevention%2Facestudy%2Ffastfact.html. Accessed 2020.<br>● CommonHealth ACTION. 2016. "Leveraging the Social Determinants to Build a Culture of Health." https://healthequity.globalpolicysolutions.org/wp-content/uploads/2016/12/RWJF_SDOH_Final_Report-002.pdf. Accessed 2020.<br>● DallaPiazza, Michelle, Mercedes Padilla-Register, Megana Dwarakanath, Elyon Obamedo, James Hill, and Maria L. Soto-Greene. 2018. "Exploring Racism and Health: An Intensive Interactive Session for Medical Students." *MedEdPORTAL*. 14:10783. https://doi.org/10.15766/mep_2374-8265.10783.<br>● Johnson, Tiffani J. 2020. "Intersection of Bias, Structural Racism, and Social Determinants with Health Care Inequities." *Pediatrics*. 146(2): e2020003657. https://doi.org/10.1542/peds.2020-003657.<br>● MedEdPORTAL. "Anti-Racism in Medicine Collection." https://www.mededportal.org/anti-racism. Accessed 2020.<br>● Trent, Maria, Danielle G. Dooley, Jacqueline Dougé, Section on Adolescent Health, Council on Community Pediatrics, Committee on Adolescence, Robert M. Cavanaugh, et al. 2019. "The Impact of Racism on Child and Adolescent Health." *Pediatrics*. 144(2):e20191765. https://doi.org/10.1542/peds.2019-1765.

**Systems-Based Practice 6: Physician Role in Health Care Systems**<br>**Overall Intent:** To understand the physician's role in health systems science to optimize patient care delivery, including cost-conscious care	
**Milestones**	**Examples**
**Level 1** *Engages with patients and other providers in discussions about cost-conscious care and key components of the health care delivery system*	● Considers the differences in cost for a patient in the hospital versus being closely followed as an outpatient<br>● Articulates the impact of patients coming to clinic for non-emergent acute visits instead of seeking care in the emergency department<br>● Considers that insurance coverage, or lack of coverage, can affect prescription drug availability/cost for individual patients<br>● Identifies that one's own implicit biases contribute to disparities and less-than-optimal care
**Level 2** *Identifies the relationships between the delivery system and cost-conscious care and the impact on the patient care*	● Considers the patient's prescription drug coverage when choosing a basal insulin<br>● Ensures that a patient hospitalized with ketosis and dehydration has a scheduled follow-up appointment at discharge
**Level 3** *Discusses the need for changes in clinical approaches based on evidence, outcomes, and cost-effectiveness to improve care for patients and families*	● Considers the need to order insulin antibodies in a young thin child with new onset diabetes<br>● Does not order thyroid levels for all patients with obesity and engages primary care practitioners in similar discussion<br>● Adapts plan to minimize costs and provides appropriate care for an uninsured patient<br>● Considers health care disparities in pursuit of evidence-based care
**Level 4** *Advocates for the promotion of safe, quality, and high-value care*	● Works collaboratively to identify additional services for a patient with a craniopharyngioma and limited resources<br>● Identifies the value of an action plan upon discharge to minimize hospital readmissions and implements a project to address this issue
**Level 5** *Coaches others to promote safe, quality, and high-value care across health care systems*	● Raises awareness at a systems level to promote cost-conscious care<br>● Leads team members in conversations around care gaps for LGBTQ+ teens and creates team plans to provide comprehensive care in a clinic<br>● Educates colleagues on local or regional food deserts and coordinates activity to address the need
Assessment Models or Tools	● Direct observation<br>● Multisource feedback<br>● Patient satisfaction data<br>● Review and guided reflection on costs accrued for individual patients or patient populations with a given diagnosis
Curriculum Mapping	●

Notes and Resources	• Agency for Healthcare Research and Quality (AHRQ). Measuring the Quality of Physician Care. https://www.ahrq.gov/talkingquality/measures/setting/physician/index.html. Accessed 2022.<br>• American Board of Pediatrics. “Entrustable Professional Activities for Subspecialties.” Pediatric Endocrinology. https://www.abp.org/content/entrustable-professional-activities-subspecialties. Accessed 2021.<br>• American College of Physicians. “Newly Revised: Curriculum for Educators and Residents (Version 4.0).” https://www.acponline.org/clinical-information/high-value-care/medical-educators-resources/newly-revised-curriculum-for-educators-and-residents-version-40. Accessed 2020.<br>• Choosing Wisely. 2017. “American Academy of Pediatrics – Section on Endocrinology: Five Things Physicians and Patients Should Question.” https://www.choosingwisely.org/societies/american-academy-of-pediatrics-section-on-endocrinology/.<br>• The Commonwealth Fund. “State Health Data Center.” http://datacenter.commonwealthfund.org/?_ga=2.110888517.1505146611.1495417431-1811932185.1495417431#ind=1/sc=1. Accessed 2020.<br>• Dzau, Victor J., Mark McClellan, Sheila Burke, Molly J. Coye, Thomas A. Daschle, Angela Diaz, William H. Frist, et al. 2017. “Vital Directions for Health and Health Care: Priorities from a National Academy of Medicine Initiative.” *NAM Perspectives*. Discussion Paper, National Academy of Medicine, Washington, DC. https://doi.org/10.31478/201703e.<br>• Solutions for Patient Safety. “Hospital Resources.” https://www.solutionsforpatientsafety.org/for-hospitals/hospital-resources/. Accessed 2020.

Practice-Based Learning and Improvement 1: Evidence-Based and Informed Practice<br>**Overall Intent:** To incorporate evidence and apply it to individual patients and patient populations	
**Milestones**	**Examples**
**Level 1** *Develops an answerable clinical question and demonstrates how to access available evidence, with guidance*	● Identifies a question such as, "What is the appropriate treatment for this patient with hyperthyroidism?", but needs guidance to focus it into a searchable question<br>● Uses general medical resources (i.e., background information) such as UpToDate or DynaMed to search for answers<br>● Accesses available evidence using unfiltered resources, retrieving a broad array of related information
**Level 2** *Independently articulates clinical question and accesses available evidence*	● Clearly identifies a focused, answerable question (e.g., "Among pre-term infants with low thyroxine (T4), does initiation of levothyroxine improve outcomes compared to observation?")<br>● Uses PubMed to search for the answer to a clinical question
**Level 3** *Locates and applies the evidence, integrated with patient preference, to the care of patients*	● Efficiently searches and filters key databases, retrieving information that is specific to the clinical question<br>● Evaluates diagnostic criteria that center around social identifiers such as race, gender, and body mass index (BMI)
**Level 4** *Critically appraises and applies evidence, even in the face of uncertainty and conflicting evidence to guide care tailored to the individual patient*	● Weighs primary and secondary outcomes to enhance specificity to individual patients<br>● Elicits patient's prior experiences regarding diversity, equity, and inclusion in the health care system to start conversations about optimal management patient preference<br>● Explores, evaluates, and incorporates new resources into search strategies<br>● Discusses with patients' families if alternative options (e.g., gonadotropin-releasing hormone analogs (GnRHa) versus observation for idiopathic central precocious puberty in a seven-year-old female) may be reasonable, while considering patient preferences/needs<br>● Uses levels of evidence to weigh the primary outcomes that apply to the care of individual patients
**Level 5** *Coaches others to critically appraise and apply evidence for complex patients*	● Provides feedback to other learners on their ability to formulate questions, search for the best available evidence, appraise evidence, and apply that information to the care of patients<br>● Participates in the development of clinical guidelines/pathways<br>● Acts as a role model and coaches others in creating efficient and effective search strategies to answer clinical questions<br>● As part of a team, develops an evidence-based clinical pathway for diabetic ketoacidosis (DKA)
Assessment Models or Tools	● Direct observation

	• Journal club and case discussions<br>• Medical record (chart) audit<br>• Presentation evaluation
Curriculum Mapping	•
Notes or Resources	• American Board of Pediatrics. "Entrustable Professional Activities for Subspecialties." Pediatric Endocrinology. https://www.abp.org/content/entrustable-professional-activities-subspecialties. Accessed 2021.<br>• Duke University. "Evidence-Based Practice." https://guides.mclibrary.duke.edu/ebm/home. Accessed 2020.<br>• Guyatt, Gordon, Drummond Rennie, Maureen O. Meade, and Deborah Cook. 2015. *Users' Guides to the Medical Literature: A Manual for Evidence-Based Clinical Practice*, 3rd ed. USA: McGraw-Hill Education. https://jamaevidence.mhmedical.com/Book.aspx?bookId=847. Accessed 2020.<br>• US National Library of Medicine. "PubMed® Online Training." https://www.nlm.nih.gov/bsd/disted/pubmedtutorial/cover.html. Accessed 2020.

Practice-Based Learning and Improvement 2: Reflective Practice and Commitment to Personal Growth<br>**Overall Intent:** To continuously improve patient care based on self-evaluation and lifelong learning	
**Milestones**	**Examples**
**Level 1** *Participates in feedback sessions*<br><br>*Develops personal and professional goals, with assistance*	● Attends scheduled feedback sessions<br><br>● Develops individualized learning plan with guidance from faculty members<br>● Acknowledges own implicit/explicit biases
**Level 2** *Demonstrates openness to feedback and performance data*<br><br>*Designs a learning plan based on established goals, feedback, and performance data, with assistance*	● Acknowledges concerns about timely note completion and works with clinic preceptor to develop goals for improvement<br><br>● Devises a plan to explore biases and how they impact care of peer relationships
**Level 3** *Seeks and incorporates feedback and performance data episodically*<br><br>*Designs and implements a learning plan by analyzing and reflecting on the factors which contribute to gap(s) between performance expectations and actual performance*	● Evaluates the ketone action plans for patients in the continuity clinic to ensure each one has an appropriate plan consistent with current guidelines<br><br>● Identifies problems in performing evaluation and management for delayed puberty and implements a plan to see more patients with this chief complaint in faculty clinic<br>● Reflects on care for a transgender male, recognizes possible implicit bias, and takes steps to mitigate bias
**Level 4** *Seeks and incorporates feedback and performance data consistently*<br><br>*Adapts a learning plan using long-term professional goals, self-reflection, and performance data to measure its effectiveness*	● Acknowledges low personal rates of microalbumin screening and initiates habits to increase rate of own screening<br>● Uses care plans to facilitate compliance with published guidelines<br><br>● Adapts learning plan to improve knowledge of office-based diabetes care based on personal reflection, feedback, and patient data<br>● Actively seeks out conferences to learn about anti-racism and bystander culture
**Level 5** *Role models and coaches others in seeking and incorporating feedback and performance data*<br><br>*Demonstrates continuous self-reflection and coaching of others on reflective practice*	● Leads a clinic discussion on opportunities to improve ketone action plan implementation for all patients with type 1 diabetes cared for by the clinic<br><br>● Meets with learners to review practice habits and develop their learning goals
Assessment Models or Tools	● Direct observation<br>● Medical record (chart) audit<br>● Review of learning plan

Curriculum Mapping	•
Notes or Resources	• American Board of Pediatrics. “Entrustable Professional Activities for Subspecialties.” Pediatric Endocrinology. https://www.abp.org/content/entrustable-professional-activities-subspecialties. Accessed 2021.<br>• Burke, Anne E., Bradley Benson, Robert Englander, Carol Carraccio, and Patricia J. Hicks. 2014. “Domain of Competence: Practice-Based Learning and Improvement.” *Academic Pediatrics.* 14(2): S38-S54. DOI: https://doi.org/10.1016/j.acap.2013.11.018.<br>• Lockspeiser, Tai M., Su-Ting T. Li, Ann E. Burke, Adam A. Rosenberg, Alston E. Dunbar 3rd, Kimberly A. Gifford, Gregory H. Gorman, et al. 2016. “In Pursuit of Meaningful Use of Learning Goals in Residency: A Qualitative Study of Pediatric Residents.” *Academic Medicine*. 91(6):839-846. DOI: 10.1097/ACM.0000000000001015.<br>• Lockspeiser, Tai M., Patricia A. Schmitter, J. Lindsey Lane, Janice L. Hanson, Adam A. Rosenberg, and Yoon Soo Park. 2013. “Assessing Residents’ Written Learning Goals and Goal Writing Skill: Validity Evidence for the Learning Goal Scoring Rubric.” *Academic Medicine*. 88(10):1558-1563. DOI: 10.1097/ACM.0b013e3182a352e6.

Professionalism 1: Professional Behavior<br>**Overall Intent:** To demonstrate ethical and professional behaviors and promote these behaviors in others, and to use appropriate resources to manage professional dilemmas	
**Milestones**	**Examples**
**Level 1** *Identifies expected professional behaviors and potential triggers for lapses*<br><br>*Identifies the value and role of pediatric endocrinology as a vocation/career*	● After receiving a consult at 3:00 a.m., asks faculty members for feedback, recognizing that they are sometimes impatient when woken up in the middle of the night<br><br>● Acknowledges the importance of pediatric endocrinologists in informing the public about healthy eating and the risk of obesity
**Level 2** *Demonstrates professional behavior with occasional lapses*<br><br>*Demonstrates accountability for patient care as a pediatric endocrinologist, with guidance*	● Is late to morning rounds, identifies this lapse, and immediately apologizes to peers and attendings upon arrival; makes an effort to be on time in the future<br><br>● When the family of a patient with type 1 diabetes asks for a letter for travel, communicates the family's request to the certified diabetes educator
**Level 3** *Maintains professional behavior in increasingly complex or stressful situations*<br><br>*Fully engages in patient care and holds oneself accountable*	● After a busy night on-call, demonstrates caring and compassionate behaviors with patients, patients' families, colleagues, and staff members<br>● Advocates for an individual patient's needs in a humanistic and professional manner regarding home care, medication approval, and need for care by another subspecialist<br>● Despite a difficult and demanding situation, continues to work to provide optimal patient care
**Level 4** *Recognizes situations that may trigger professionalism lapses and intervenes to prevent lapses in self and others*<br><br>*Exhibits a sense of duty to patient care and professional responsibilities*	● Models respect and compassion for patients and promotes the same from colleagues by actively identifying positive professional behavior<br><br>● Without prompting, assists colleagues with seeing patients when the clinic is busy<br>● Speaks up in the moment when observing racist/sexist behavior within the health care team and uses reporting mechanisms to address it
**Level 5** *Models professional behavior and coaches others when their behavior fails to meet professional expectations*<br><br>*Extends the role of the pediatric endocrinologist beyond the care of patients by engaging with the community, specialty, and medical profession as a whole*	● Discusses the need to be on time with a PGY-4 who continues to be late, plans together to address the underlying issues of why the learner is late<br><br>● Advocates for process improvement to prevent hypoglycemia in hospitalized patients treated with insulin<br>● Develops education and/or modules on microaggressions and bias
Assessment Models or Tools	● Direct observation<br>● Global evaluation

	• Lectures and workshops about professionalism<br>• Multisource feedback<br>• Oral or written self-reflection
Curriculum Mapping	•
Notes or Resources	• AbdelHameid, Duaa. 2020. "Professionalism 101 for Black Physicians." New England Journal of Medicine. 383(5): e34. doi:10.1056/NEJMpv2022773.<br>• American Academy of Pediatrics. "Residency Curriculum Mental Health Education Resources." https://www.aap.org/en-us/advocacy-and-policy/aap-health-initiatives/Mental-Health/Pages/Residency-Curriculum.aspx. Accessed 2020.<br>• American Board of Internal Medicine Foundation, ACP-ASIM Foundation, and European Federation of Internal Medicine. 2002. "Medical Professionalism in the New Millennium: A Physician Charter." *Annals of Internal Medicine* 136: 243-246. https://doi.org/10.7326/0003-4819-136-3-200202050-00012.<br>• American Board of Pediatrics. "Entrustable Professional Activities for Subspecialties." Pediatric Endocrinology. https://www.abp.org/content/entrustable-professional-activities-subspecialties. Accessed 2021.<br>• American Board of Pediatrics. "Medical Professionalism." https://www.abp.org/content/medical-professionalism. Accessed 2020.<br>• American Board of Pediatrics. "Teaching, Promoting, and Assessing Professionalism Across the Continuum: A Medical Educator's Guide." https://www.abp.org/professionalism-guide. Accessed 2020.<br>• American Medical Association. "Ethics." https://www.ama-assn.org/delivering-care/ama-code-medical-ethics. Accessed 2020.<br>• Bynny, Richard L., Douglas S. Paauw, Maxine Papadakis, and Sheryl Pfeil. 2017. *Medical Professionalism Best Practices: Professionalism in the Modern Era*. Aurora, CO: Alpha Omega Alpha Medical Society. https://www.alphaomegaalpha.org/wp-content/uploads/2022/01/Monograph2018.pdf. ISBN: 978-1-5323-6516-4.<br>• Domen, Ronald E., Kristen Johnson, Richard Michael Conran, Robert D. Hoffman, Miriam D. Post, Jacob J. Steinberg, Mark D. Brissette, et al. 2016. "Professionalism in Pathology: A Case-Based Approach as a Potential Educational Tool." *Archives of Pathology and Laboratory Medicine* 141: 215-219. https://doi.org/10.5858/arpa.2016-0217-CP.<br>• Levinson, Wendy, Shiphra Ginsburg, Frederic W. Hafferty, and Catherine R. Lucey. 2014. *Understanding Medical Professionalism*. New York, NY: McGraw-Hill Education. https://accessmedicine.mhmedical.com/book.aspx?bookID=1058.<br>• Osseo-Asare, Aba, Lilanthi Balasuriya, Stephen J. Huot, et al. 2018. "Minority Resident Physicians' Views on the Role of Race/Ethnicity in Their Training Experiences in the

	Workplace." *JAMA Network Open*. 1(5): e182723. doi:10.1001/jamanetworkopen.2018.2723.<br>● Paul, Dereck W. Jr., Kelly R. Knight, Andre Campbell, and Louise Aronson. 2020. "Beyond a Moment - Reckoning with Our History and Embracing Antiracism in Medicine." *New England Journal of Medicine*. 383: 1404-1406. doi:10.1056/NEJMp2021812 https://www.nejm.org/doi/full/10.1056/NEJMp2021812.

**Professionalism 2: Ethical Principles**<br>**Overall Intent:** To recognize and address or resolve common and complex ethical dilemmas or situations	
**Milestones**	**Examples**
**Level 1** *Demonstrates knowledge of the ethical principles underlying informed consent, surrogate decision making, advance directives, confidentiality, error disclosure, stewardship of limited resources, and related topics*	● Identifies and applies ethical principles involved in informed consent in genetic testing<br>● Identifies medical errors and the need to disclose them
**Level 2** *Applies ethical principles in common situations*	● Demonstrates understanding that while additional testing may be educational, it may not provide clinical benefit to the patient
**Level 3** *Analyzes complex situations using ethical principles to address conflict/controversy; seeks help when needed to manage and resolve complex ethical situations*	● Offers treatment options for a patient with type 1 diabetes, minimizing bias, while recognizing own limitations, and consistently honoring the patient's and patient's family's choice<br>● Provides support to a young mother who has custody of her daughter, while team discusses potential custody issues; rationally and calmly discusses conflict regarding custody with attending<br>● Recognizes that prior experiences of racism for the patient and patient's family influence their trust, and defers discussion of the most complex issues to the care team member(s) in whom the family members have demonstrated trust, rather than assuming a hierarchical structure
**Level 4** *Manages and seeks to resolve ethical dilemmas using appropriate resources (e.g., ethics consultations, literature review, risk management/legal consultation)*	● Uses institutional resources, including social work and risk management, when a parent chooses to leave the hospital against medical advice<br>● Engages with a multidisciplinary team to address issues in the management of a patient with suboptimal diabetes control
**Level 5** *Called upon by others to consult in cases of complex ethical dilemmas; identifies and seeks to address system-level factors that induce or exacerbate*	● Lobbies insurance companies to provide coverage for diabetes technology<br>● Advocates for affordable insulin
Assessment Models or Tools	● Direct observation<br>● Global evaluation<br>● Lectures and workshops about professionalism<br>● Multisource feedback<br>● Oral or written self-reflection
Curriculum Mapping	●

Notes or Resources	• American Board of Pediatrics. "Entrustable Professional Activities for Subspecialties." Pediatric Endocrinology. https://www.abp.org/content/entrustable-professional-activities-subspecialties. Accessed 2021.<br>• American Medical Association. "Ethics." https://www.ama-assn.org/delivering-care/ama-code-medical-ethics. Accessed 2020.<br>• Bynny, Richard L., Douglas S. Paauw, Maxine Papadakis, and Sheryl Pfeil. 2017. *Medical Professionalism Best Practices: Professionalism in the Modern Era*. Aurora, CO: Alpha Omega Alpha Medical Society. https://www.alphaomegaalpha.org/wp-content/uploads/2022/01/Monograph2018.pdf. ISBN: 978-1-5323-6516-4.<br>• Fallat, Mary E., Jacqueline Glover, American Academy of Pediatrics, Committee on Bioethics. 2007. "Professionalism in Pediatrics." *Pediatrics* 120(4): e1123–e1133. https://doi.org/10.1542/peds.2007-2230.<br>• Levinson, Wendy, Shiphra Ginsburg, Frederic W. Hafferty, and Catherine R. Lucey. 2014. *Understanding Medical Professionalism*. New York, NY: McGraw-Hill Education. https://accessmedicine.mhmedical.com/book.aspx?bookID=1058.

**Professionalism 3: Accountability/Conscientiousness**<br>**Overall Intent:** To take responsibility for one's own actions and the impact on patients and other members of the health care team	
**Milestones**	**Examples**
**Level 1** *Performs tasks and responsibilities, with prompting*	● Responds to reminders from program administrator to complete work hour logs<br>● After being informed by the program director that too many conferences have been missed, changes habits to meet the minimum attendance requirement<br>● Completes patient care tasks (callbacks, consultations, orders) after prompting from a supervisor
**Level 2** *Performs tasks and responsibilities in a timely manner in routine situations*	● Completes administrative tasks (e.g., licensing requirements) by specified due date<br>● Completes routine patient care tasks as assigned<br>● Answers pages and emails promptly with rare need for reminders
**Level 3** *Performs tasks and responsibilities in a thorough and timely manner in complex or stressful situations*	● Identifies multiple competing demands when caring for patients, appropriately triages tasks, and appropriately seeks help from other team members
**Level 4** *Coaches others to ensure tasks and responsibilities are completed in a thorough and timely manner in complex or stressful situations*	● Provides other fellows with tips on task prioritization<br>● Guides the primary team to ensure that all tasks are completed for safe and thorough patient care
**Level 5** *Creates strategies to enhance others' ability to efficiently complete tasks and responsibilities*	● Meets with multidisciplinary team (e.g., nurses, social worker, case manager) to streamline patient discharges
Assessment Models or Tools	● Compliance with deadlines and timelines<br>● Direct observation<br>● Global evaluations<br>● Multisource feedback<br>● Self-evaluations and reflective tools
Curriculum Mapping	●
Notes or Resources	● American Board of Pediatrics. "Entrustable Professional Activities for Subspecialties." Pediatric Endocrinology. https://www.abp.org/content/entrustable-professional-activities-subspecialties. Accessed 2021.<br>● American Medical Association. "Ethics." https://www.ama-assn.org/delivering-care/ama-code-medical-ethics. Accessed 2020.<br>● Bynny, Richard L., Douglas S. Paauw, Maxine Papadakis, and Sheryl Pfeil. 2017. *Medical Professionalism Best Practices: Professionalism in the Modern Era*. Aurora, CO: Alpha Omega Alpha Medical Society. https://www.alphaomegaalpha.org/wp-content/uploads/2022/01/Monograph2018.pdf. ISBN: 978-1-5323-6516-4.

	• Fallat, Mary E., Jacqueline Glover, American Academy of Pediatrics, Committee on Bioethics. 2007. “Professionalism in Pediatrics.” *Pediatrics* 120(4): e1123–e1133. https://doi.org/10.1542/peds.2007-2230.<br>• Frohna, John G., and Jamie S. Padmore. 2021. “Assessment of Professionalism in the Graduate Medical Education Environment.” *Journal of Graduate Medical Education* 13(2 Suppl): 81-85. https://doi.org/10.4300/JGME-D-20-00845.1. PMID: 33936538 PMCID: PMC8078080.<br>• Hodges, Brian David, Shiphra Ginsburg, Richard Cruess, Sylvia Cruess, Rhena Delport, Fred Hafferty, Ming-Jung Ho, et al. 2011. “Assessment of Professionalism: Recommendations from the Ottawa 2010 Conference.” *Medical Teacher* 33(5): 354-63. doi: 10.3109/0142159X.2011.577300.<br>• Levinson, Wendy, Shiphra Ginsburg, Frederic W. Hafferty, and Catherine R. Lucey. 2014. *Understanding Medical Professionalism*. New York, NY: McGraw-Hill Education. https://accessmedicine.mhmedical.com/book.aspx?bookID=1058.<br>• Code of conduct from fellow/resident institutional manual<br>• Expectations of residency program regarding accountability and professionalism

**Professionalism 4: Well-Being**<br>**Overall Intent:** To identify resources to manage and improve well-being	
**Milestones**	**Examples**
**Level 1** *Recognizes the importance of addressing personal and professional well-being*	● Discusses the importance of a faculty mentor<br>● Recognizes that personal stress may require asking for help from the training program
**Level 2** *Describes institutional resources that are meant to promote well-being*	● Identifies well-being resources such as meditation apps, mental health resources, or spiritual or religious resources for learners available through the program and institution<br>● Meets with program director to discuss Family Medical Leave Act options when expecting a child
**Level 3** *Recognizes institutional and personal factors that impact well-being*	● Identifies that working on a consult service may be stressful and impact well-being<br>● Describes the tension between professional and personal responsibilities
**Level 4** *Describes interactions between institutional and personal factors that impact well-being*	● Discusses a plan to mitigate the tension between a busy schedule and time with family<br>● Recognizes how microaggressions from coworkers and/or faculty members are impacting performance or engagement in patient care
**Level 5** *Coaches and supports colleagues to optimize well-being at the team, program, or institutional level*	● Leads organizational efforts to address clinician well-being<br>● Develops an affinity group to provide support for self and others to explore impact of microaggressions and biases
Assessment Models or Tools	● Direct observation<br>● Group interview or discussions for team activities<br>● Individual interview<br>● Institutional online training modules<br>● Self-assessment and personal learning plan
Curriculum Mapping	●
Notes or Resources	● This subcompetency is not intended to evaluate a fellow's well-being, but to ensure each fellow has the fundamental knowledge of factors that impact well-being, the mechanisms by which those factors impact well-being, and available resources and tools to improve well-being.<br>● Local resources, including employee assistance programs<br>● ACGME. "Well-Being." https://dl.acgme.org/pages/well-being-tools-resources. Accessed 2022.<br>● Aggarwal, Rashi, Jill K. Deutsch, Jose Medina, and Neil Kothari. 2017. "Resident Wellness: An Intervention to Decrease Burnout and Increase Resiliency and Happiness." *MedEdPORTAL*. 13:10651. doi:10.15766/mep_2374-8265.10651.

	• American Board of Pediatrics. "Entrustable Professional Activities for Subspecialties." Pediatric Endocrinology. https://www.abp.org/content/entrustable-professional-activities-subspecialties. Accessed 2021.<br>• Bayer, Nathaniel D., and Peter S. Capucilli. 2018. "Small Steps to Address Medical Resident Burnout." *JAMA Pediatrics* 172(2):113-114. doi:10.1001/jamapediatrics.2017.4166.<br>• Eckleberry-Hunt, Jodie, Anne Van Dyke, David Lick, and Jennifer Tucciarone. 2009. "Changing the Conversation from Burnout to Wellness: Physician Well-Being in Residency Training Programs." *Journal of Graduate Medical Education* (2): 225–230. doi:10.4300/JGME-D-09-00026.1.<br>• Hicks, Patricia J., Daniel Schumacher, Susan Guralnick, Carol Carraccio, and Ann E. Burke. 2014. "Domain of Competence: Personal and Professional Development." *Academic Pediatrics*. 14(2 Suppl): S80-97. https://doi.org/10.1016/j.acap.2013.11.017.<br>• Mendoza, Dexter, Anna Holbrook, Frederic Bertino, David Theriot, and Christopher Ho. 2019. "Using Wellness Days to Mitigate Resident Burnout." *Journal of the American College of Radiology* 16(2): 221-223. doi:10.1016/j.jacr.2018.09.005.<br>• Raj, Kristin S. 2016. "Well-Being in Residency: A Systematic Review." *Journal of Graduate Medical Education* 8(5): 674-684. doi:10.4300/JGME-D-15-00764.1.<br>• Ripp, Jonathan A., Michael R. Privitera, Colin P. West, Richard Leiter, Lia Logio, Jo Shapiro, and Hasan Bazari. 2017. "Well-Being in Graduate Medical Education: A Call for Action." *Academic Medicine* 92(7): 914-917. doi: 10.1097/ACM.0000000000001735.<br>• Sklar, David P. 2016. "Fostering Student, Resident, and Faculty Wellness to Produce Healthy Doctors and a Healthy Population." *Academic Medicine* 91(9): 1185–1188. doi:10.1097/ACM.0000000000001298.<br>• Tawfik, Daniel S., Jochen Profit, Timothy I. Morgenthaler, Daniel V. Satele, Christine A. Sinsky, Liselotte N. Dyrbye, Michael A. Tutty, Colin P. West, and Tait D. Shanafelt. 2018. "Physician Burnout, Well-Being, and Work Unit Safety Grades in Relationship to Reported Medical Errors." *Mayo Clinical Proceedings* 93(11): 1571-1580. doi:10.1016/j.mayocp.2018.05.014.

Interpersonal and Communication Skills 1: Patient- and Family-Centered Communication<br>**Overall Intent:** To establish a therapeutic relationship with patients and their families, tailor communication to the needs of patients and patients' families, and effectively navigate difficult/sensitive conversations	
**Milestones**	**Examples**
**Level 1** *Demonstrates respect and attempts to establish rapport*<br><br>*Attempts to adjust communication strategies based upon patient/family expectations*	● Introduces self and faculty member, identifies patient and others in the room, and engages all parties in health care discussion<br>● Acknowledges the need to initiate a sensitive conversation and determines appropriate health care practitioner for the conversation<br><br>● Focuses visit on patient and parental/caregiver concerns
**Level 2** *Establishes a therapeutic relationship in straightforward encounters*<br><br>*Adjusts communication strategies as needed to mitigate barriers and meet patient/family expectations*	● Prioritizes and sets an agenda based on concerns of patient's parents/caregivers at the beginning of a health supervision visit with a child with an acute or chronic medical problem<br>● Uses nonjudgmental language to discuss sensitive topics<br>● Uses patient's preferred pronouns when addressing patient<br><br>● When seeing a distraught teenager with diabetes, ensures the patient understands that with proper glycemic control, long-term complications may be avoided<br>● Identifies the need for a medical interpreter when evaluating a non-English-speaking patient and/or family
**Level 3** *Establishes a culturally competent and therapeutic relationship in most encounters*<br><br>*Communicates with sensitivity and compassion, elicits patient/family values, and acknowledges uncertainty and conflict*	● Prioritizes and sets an agenda based on concerns of patient's parents/caregivers at the beginning of a health supervision visit with a child with multiple chronic medical problems<br>● Discusses sensitive topics while promoting trust, respect, and understanding<br>● Recognizes that mispronouncing a patient's name, especially one of a different ethnicity, might be experienced as a microaggression; apologizes to the patient and seeks to correct the mistake
**Level 4** *Establishes a therapeutic relationship in straightforward and complex encounters, including those with ambiguity and/or conflict*<br><br>*Uses shared decision making with patient/family to make a personalized care plan*	● Continues to engage parents/caregivers who insist on growth hormone treatment when not clinically indicated, addressing misinformation and reviewing risks/benefits to assuage these concerns in a manner that engages rather than alienates the patient's family<br>● Facilitates sensitive discussions with the patient/family and interdisciplinary team<br>● Asks questions in ways that validate patient identity and promote an inclusive environment<br>● While maintaining trust, engages family of a child with medical complexity along with other members of the multi-specialty care team in determining the family's wishes and expectations regarding gender of rearing in a child with disorder of sex development

**Level 5** *Mentors others to develop positive therapeutic relationships*<br><br>*Models and coaches others in patient- and family-centered communication*	● Acts as a mentor for a resident discussing diagnosis of type 1 diabetes to a patient and the patient's family<br>● Mentors a junior learner to facilitate a conversation with the patient's family members and multidisciplinary team in a patient with disorder of sex development<br><br>● Develops a curriculum on patient- and family-centered communication, including navigating difficult conversations
Assessment Models or Tools	● Direct observation<br>● Kalamazoo Essential Elements Communication Checklist (Adapted)<br>● Skills needed to Set the state, Elicit information, Give information, Understand the patient, and End the encounter (SEGUE)
Curriculum Mapping	●
Notes or Resources	● American Board of Pediatrics. "Entrustable Professional Activities for Subspecialties." Pediatric Endocrinology. https://www.abp.org/content/entrustable-professional-activities-subspecialties. Accessed 2021.<br>● Benson, Bradley J. 2014. "Domain of Competence: Interpersonal and Communication Skills." *Academic Pediatrics* 14(2 Suppl):S55-S65. https://doi.org/10.1016/j.acap.2013.11.016.<br>● Laidlaw, Anita, and Jo Hart. 2011. "Communication Skills: An Essential Component of Medical Curricula. Part I: Assessment of Clinical Communication: AMEE Guide No. 51." *Medical Teacher*. 33(1): 6-8. https://doi.org/10.3109/0142159X.2011.531170.<br>● Makoul, Gregory. 2001. "Essential Elements of Communication in Medical Encounters: the Kalamazoo Consensus Statement." *Academic Medicine*. 76(4): 390-393. https://journals.lww.com/academicmedicine/Fulltext/2001/04000/Essential_Elements_of_Communication_in_Medical.21.aspx#pdf-link.<br>● Makoul, Gregory. 2001. "The SEGUE Framework for Teaching and Assessing Communication Skills." *Patient Education and Counseling*. 45(1): 23-34. https://doi.org/10.1016/S0738-3991(01)00136-7.<br>● MedEdPORTAL. "Anti-Racism in Medicine Collection." https://www.mededportal.org/anti-racism. Accessed 2020.<br>● National LGBTQIA+ Health and Education Center: https://www.lgbtqiahealtheducation.org/.

**Interpersonal and Communication Skills 2: Patient and Family Education**<br>**Overall Intent:** To effectively educate patients and use shared decision making to improve outcomes	
**Milestones**	**Examples**
**Level 1** *Recognizes link between patient outcomes and education*	● Recognizes that patients should understand their diagnosis of diabetes and the importance of taking their medication to prevent diabetic ketoacidosis
**Level 2** *Identifies styles and practices for effective patient education and the importance of a team approach*	● Is aware that patient education handouts might not be the correct method of education for all patients' families<br>● Teaches a resident the importance of incorporating the dietitian, diabetes educator, and psychologist in the education of a patient with new onset diabetes and the patient's family
**Level 3** *Educates patients using defined scripts and non-targeted methods*	● Memorizes a script when discussing a new diagnosis of diabetes with a patient and the patient's family without adjusting for the family's level of understanding
**Level 4** *Tailors education to individual patients' and their families' needs by using varying scripts and methods*	● For a patient with new onset diabetes, focuses education on current patient/family needs without sticking to a script<br>● Checks for patient/family level of understanding following discussion to provide additional information as needed<br>● Provides a treatment schedule chart for the treatment of adrenal insufficiency for a patient's family with a low level of understanding<br>● Educates a 10-year-old patient with developmentally appropriate information regarding the patient's diagnosis of congenital adrenal hyperplasia and the need for on-going care
**Level 5** *Educates patients in self-advocacy and available community resources*	● Encourages caregivers to communicate with school administration about obtaining a 504 plan and accommodations for their child with endocrine disease
Assessment Models or Tools	● Direct observation<br>● Case discussions<br>● Multisource feedback<br>● Self-assessment<br>● Self-reflection
Curriculum Mapping	●
Notes or Resources	● Jotterand, Fabrice, Antonio Amodio, and Bernice S. Elger. 2016. "Patient Education as Empowerment and Self-Rebiasing." *Medicine, Health Care and Philosophy* 19(4): 553-561. https://doi.org/10.1007/s11019-016-9702-9.<br>● Lindeman, C.A. 1988. "Patient Education." *Annual Review of Nursing Research*. 6:29-60.<br>● Parent, Kelly, Kori Jones, Lauren Phillips, Jennifer N. Stojan, and Joseph B. House. 2016. "Teaching Patient- and Family-Centered Care: Integrating Shared Humanity into Medical Education Curricula. *AMA Journal of Ethics* 18(1):24-32. https://doi.org/10.1001/journalofethics.2016.18.1.medu1-1601.

	• Vital Talks

Interpersonal and Communication Skills 3: Interprofessional and Team Communication<br>**Overall Intent:** To communicate effectively with the health care team, including consultants	
**Milestones**	**Examples**
**Level 1** *Respectfully requests a consultation, with guidance*<br><br>*Identifies the members of the interprofessional team*	• When asking for a cardiology consultation for a patient with Turner syndrome, respectfully relays the diagnosis and requests for the team to evaluate the patient<br><br>• Acknowledges the contribution of each member of the multidisciplinary team to the patient
**Level 2** *Clearly and concisely requests consultation by communicating patient information*<br><br>*Participates within the interprofessional team*	• When requesting a consultation from the cardiology team, clearly and concisely describes the recent history of a patient diagnosed with Turner syndrome and found to have a murmur<br><br>• Sends a message in the EHR to the dietitian to discuss water requirements for a child with diabetes insipidus
**Level 3** *Formulates a specific question for consultation and tailors communication strategy*<br><br>*Uses bi-directional communication within the interprofessional team*	• When requesting a consultation for the cardiology team for a patient with Turner syndrome, specifically asks for the need for echocardiogram<br><br>• Contacts the endocrinology social worker to arrange for delivery of a specialized formula and completes the prescription as requested by the social worker<br>• Asks other members of the health care team to repeat back recommendations to ensure understanding
**Level 4** *Coordinates consultant recommendations to optimize patient care*<br><br>*Facilitates interprofessional team communication*	• After cardiology consultation is complete for a patient with Turner syndrome, reviews recommendations with the care team and patient's family and ensures recommendations are implemented<br><br>• Initiates a multidisciplinary meeting to develop shared care plan for a patient with 22q11.2 deletion syndrome<br>• Explains to the rest of the team, as well as the patient's parents/caregivers, the rationale for chromosome analysis instead of chromosome microarray analysis as the preferred diagnostic test for suspected Turner syndrome<br>• When hearing racial discrimination or microaggressions from a colleague regarding a patient with recurrent DKA, engages the colleague in a conversation about these statements
**Level 5** *Maintains a collaborative relationship with referring providers that maximizes adherence to practice recommendations*	• Collaborates with primary care practitioner to ensure a patient with Turner syndrome receives recommended screening tests when patient has missed endocrinology visit

	• Works with primary care practitioner to ensure appropriate referral to other subspecialists for a patient with Prader-Willi syndrome<br>• Works with primary care practitioner to ensure care for a patient living far from clinic who cannot easily attend appointments
Coaches others in effective communication within the interprofessional team	• Mediates a conflict among members of the health care team
Assessment Models or Tools	• Direct observation<br>• Global assessment<br>• Medical record (chart) audit<br>• Multi-source feedback
Curriculum Mapping	•
Notes or Resources	• American Board of Pediatrics. "Entrustable Professional Activities for Subspecialties." Pediatric Endocrinology. https://www.abp.org/content/entrustable-professional-activities-subspecialties. Accessed 2021.<br>• ACAPT. "NIPEC Assessment Resources and Tools." https://acapt.org/about/consortium/national-interprofessional-education-consortium-(nipec)/nipec-assessment-resources-and-tools. Accessed 2020.<br>• Dehon, Erin, Kimberly Simpson, David Fowler, and Alan Jones. 2015. "Development of the Faculty 360." *MedEdPORTAL*. 11:10174. http://doi.org/10.15766/mep_2374-8265.10174.<br>• Fay, David, Michael Mazzone, Linda Douglas, and Bruce Ambuel. 2007. "A Validated, Behavior-Based Evaluation Instrument for Family Medicine Residents." *MedEdPORTAL*. https://doi.org/10.15766/mep_2374-8265.622.<br>• François, José. 2011. "Tool to Assess the Quality of Consultation and Referral Request Letters in Family Medicine." *Canadian Family Physician*. 57(5): 574-575. https://www.ncbi.nlm.nih.gov/pmc/articles/PMC3093595/.<br>• Green, Matt, Teresa Parrott, and Graham Cook. 2012. "Improving Your Communication Skills." *BMJ*. 344:e357. https://doi.org/10.1136/bmj.e357.<br>• Henry, Stephen G., Eric S. Holmboe, and Richard M. Frankel. 2013. "Evidence-Based Competencies for Improving Communication Skills in Graduate Medical Education: A Review with Suggestions for Implementation." *Medical Teacher*. 35(5):395-403. https://doi.org/10.3109/0142159X.2013.769677.<br>• Interprofessional Education Collaborative Expert Panel. 2011. "Core Competencies for Interprofessional Collaborative Practice: Report of an Expert Panel." Washington, D.C.: Interprofessional Education Collaborative. https://www.aacom.org/docs/default-source/insideome/ccrpt05-10-11.pdf?sfvrsn=77937f97_2.

	• Roth, Christine G., Karen W. Eldin, Vijayalakshmi Padmanabhan, and Ellen M. Freidman. 2018. "Twelve Tips for the Introduction of Emotional Intelligence in Medical Education." *Medical Teacher* 41(7): 1-4. https://doi.org/10.1080/0142159X.2018.1481499.

**Interpersonal and Communication Skills 4: Communication within Health Care Systems**<br>**Overall Intent:** To effectively communicate using a variety of tools and methods	
**Milestones**	**Examples**
**Level 1** *Records accurate information in the patient record*<br><br>*Identifies the importance of and responds to multiple forms of communication (e.g., in-person, electronic health record (EHR), telephone, email)*	● Corrects progress note after attending identifies outdated plan<br>● If using copy/paste/forward in the EHR, goes back to make changes to note after doing so<br><br>● Responds to questions from other practitioners in the EHR
**Level 2** *Records accurate and timely information in the patient record*<br><br>*Selects appropriate method of communication, with prompting*	● Provides organized and accurate documentation that supports the treatment plan<br>● Completes progress notes in the expected time frame of the institution<br>● Avoids biased or stigmatized language in notes<br><br>● Calls, as opposed to using secure messaging, resident and nurse with urgent request for laboratory tests after supervising attending reminds them<br>● Communicates with support staff members when additional information is needed for a clinic appointment
**Level 3** *Concisely documents updated, prioritized, diagnostic and therapeutic reasoning in the patient record*<br><br>*Aligns type of communication with message to be delivered (e.g., direct and indirect) based on urgency and complexity*	● Provides organized and accurate documentation that supports the treatment plan and limits extraneous information<br>● Produces documentation that reflects complex clinical thinking and planning and is concise, but may not contain contingency planning (i.e., if/then statements)<br><br>● When a patient with diabetes is noted to have Kussmaul breathing at a clinic visit, immediately arranges for transport to the emergency department and calls the emergency department physician(s) to make them aware of the patient<br>● Sends EHR message to patient's cardiologist with non-urgent question rather than paging cardiologist on call
**Level 4** *Documents diagnostic and therapeutic reasoning, including anticipatory guidance*<br><br>*Demonstrates exemplary written and verbal communication*	● Produces documentation that is consistently accurate, organized, and concise; reflects complex clinical reasoning and frequently incorporates contingency planning<br><br>● Communicates effectively and proactively with collaborating physicians and teams about communication gaps to prevent recurrence
**Level 5** *Models and coaches others in documenting diagnostic and therapeutic reasoning*	● Is identified as a peer resource for modeling a range of effective tools and methods of communication that fit the context of a broad variety of clinical encounters

Coaches others in written and verbal communication	• Designs and facilitates the improvement of systems that integrates effective communication among teams, departments, and institutions<br>• Leads a team to discuss implementation and dissemination of preferred pronouns/names into EHR
Assessment Models or Tools	• Direct observation<br>• Medical record (chart) audit<br>• Multisource feedback
Curriculum Mapping	•
Notes or Resources	• American Board of Pediatrics. "Entrustable Professional Activities for Subspecialties." Pediatric Endocrinology. https://www.abp.org/content/entrustable-professional-activities-subspecialties. Accessed 2021.<br>• Benson, Bradley J. 2014. "Domain of Competence: Interpersonal and Communication Skills." *Academic Pediatrics* 14(2 Suppl):S55-S65. https://doi.org/10.1016/j.acap.2013.11.016.<br>• Bierman, Jennifer A., Kathryn Kinner Hufmeyer, David T. Liss, A. Charlotta Weaver, and Heather L. Heiman. 2017. "Promoting Responsible Electronic Documentation: Validity Evidence for a Checklist to Assess Progress Notes in the Electronic Health Record." *Teaching and Learning in Medicine.* 29(4): 420-432. https://doi.org/10.1080/10401334.2017.1303385.<br>• Haig, Kathleen M., Staci Sutton, and John Whittington. 2006. "SBAR: A Shared Mental Model for Improving Communications Between Clinicians." *Joint Commission Journal on Quality and Patient Safety.* 32(3):167-75. https://doi.org/10.1016/s1553-7250(06)32022-3.<br>• Starmer, Amy J., Nancy D. Spector, Rajendu Srivastava, April D. Allen, Christopher P. Landrigan, Theodore Sectish, and I-PASS Study Group. 2012. "I-Pass, a Mnemonic to Standardize Verbal Handoffs." *Pediatrics* 129.2:201-204. https://doi.org/10.1542/peds.2011-2966.

To help programs transition to the new version of the Milestones, the ACGME has mapped the original Milestones 1.0 to the new Milestones 2.0. Indicated below are the subcompetencies that are similar between versions. These are not exact matches, but are areas that include similar elements. Not all subcompetencies map between versions. Inclusion or exclusion of any subcompetency does not change the educational value or impact on curriculum or assessment.

Milestones 1.0	Milestones 2.0
PC1: Provide transfer of care that ensures seamless transitions	SBP4: System Navigation for Patient-Centered Care – Transitions in Care
PC2: Make informed diagnostic and therapeutic decisions that result in optimal clinical judgement	PC1: History<br>PC2: Physical Exam<br>PC4: Diagnostic Testing, Including Labs, Imaging, and Functional Testing<br>MK2: Clinical Reasoning<br>MK3: Therapeutics (Behavioral, Medications, Technology, Radiopharmaceuticals)
PC3: Develop and carry out management plans	PC3: Patient Management<br>PC5: Clinical Consultation<br>ICS1: Patient- and Family-Centered Communication
PC4: Provide appropriate role modeling	PBLI2: Reflective Practice and Commitment to Personal Growth
MK1: Locate, appraise, and assimilate evidence from scientific studies related to their patients' health problems	MK1: Physiology and Pathophysiology<br>PBLI1: Evidence Based and Informed Practice
SBP1: Work effectively in various health care delivery settings and systems relevant to their clinical specialty	SBP3: System Navigation for Patient Cantered Care – Coordination of Care<br>SBP6: Physician Role in Health Care Systems
SBP2: Coordinate patient care within the health care system relevant to their clinical specialty	SBP3: System Navigation for Patient Centered Care – Coordination of Care<br>SBP4: System Navigation for Patient-Centered Care – Transitions in Care<br>SBP5: Population and Community Health<br>ICS1: Patient- and Family-Centered Communications<br>ICS3: Interprofessional and Team Communication
SBP3: Incorporate considerations of cost awareness and risk-benefit analysis in patient and/or population-based care as appropriate	SBP5: Population and Community Health<br>SBP6: Physician Role in Health Care Systems
SBP4: Work in inter-professional teams to enhance patient safety and improve patient care quality	SBP1: Patient Safety<br>ICS3: Interprofessional and Team Communication

SBP5: Participate in identifying system errors and implementing potential systems solutions	SBP1: Patient Safety<br>SBP2: Quality Improvement
PBLI1: Identifying strengths, deficiencies, and limits to one's knowledge and expertise	PBLI1: Evidence Based and Informed Practice<br>PBLI2: Reflective Practice and Commitment to Personal Growth
PBLI2: Systematically analyze practice using quality improvement methods, and implement changes with the goal of practice improvement	SBP2: Quality Improvement<br>PBLI2: Reflective Practice and Commitment to Personal Growth
PBLI3: Use information technology to optimize learning and care delivery	PBLI1: Evidence Based and Informed Practice<br>PBLI2: Reflective Practice and Commitment to Personal Growth<br>ICS4: Communication within Health Care Systems
PBLI4: Participate in the education of patients, families, students, residents, fellows, and other health professionals	SBP5: Population and Community Health<br>PBLI1: Evidence Based and Informed Practice<br>ICS1: Patient- and Family-Centered Communications<br>ICS2: Patient and Family Education
PROF1: Professional Conduct: High standards of ethical behavior which includes maintaining appropriate professional boundaries	PROF1: Professional Behavior<br>PROF2: Ethical Principles
PROF2: Trustworthiness that makes colleagues feel secure when one is responsible for the care of patients	PBLI1: Evidence Based and Informed Practice<br>PROF1: Professional Behavior<br>PROF3: Accountability/Conscientiousness<br>ICS1: Patient- and Family-Centered Communications
PROF3: Provide leadership skills that enhance team functioning, the learning environment, and/or the health care delivery system/environment with the ultimate intent of improving care of patients	ICS3: Interprofessional and Team Communication<br>ICS4: Communication within Health Care Systems<br>PROF2: Ethical Principles<br>PROF3: Accountability/Conscientiousness
PROF4: The capacity to accept that ambiguity is part of clinical medicine and to recognize the need for and to utilize appropriate resources in dealing with uncertainty	PROF2: Ethical Principles<br>ICS1: Patient- and Family-Centered Communication<br>PBLI1: Evidence Based and Informed Practice
	PROF4: Well-Being
ICS1: Communicate effectively with physicians, other health professionals, and health-related agencies	ICS2: Interprofessional and Team Communication<br>ICS3: Communication within Health Care Systems
ICS2: Work effectively as a member or leader of a health care team or other professional group	ICS2: Interprofessional and Team Communication<br>PBLI2: Reflective Practice and Commitment to Personal Growth<br>PROF3: Accountability/Conscientiousness
ICS3: Act in a consultative role to other physicians and health professionals	PC5: Clinical Consultation<br>MK2: Clinical Reasoning

	ICS2: Interprofessional and Team Communication<br>ICS3: Communication within Health Care Systems

Available Milestones Resources

Milestones 2.0: Assessment, Implementation, and Clinical Competency Committees Supplement, new 2021 - https://meridian.allenpress.com/jgme/issue/13/2s

Clinical Competency Committee Guidebook, updated 2020 - https://www.acgme.org/Portals/0/ACGMEClinicalCompetencyCommitteeGuidebook.pdf?ver=2020-04-16-121941-380

Clinical Competency Committee Guidebook Executive Summaries, new 2020 - https://www.acgme.org/What-We-Do/Accreditation/Milestones/Resources - Guidebooks - Clinical Competency Committee Guidebook Executive Summaries

Milestones Guidebook, updated 2020 - https://www.acgme.org/Portals/0/MilestonesGuidebook.pdf?ver=2020-06-11-100958-330

Milestones Guidebook for Residents and Fellows, updated 2020 - https://www.acgme.org/Portals/0/PDFs/Milestones/MilestonesGuidebookforResidentsFellows.pdf?ver=2020-05-08-150234-750

Milestones for Residents and Fellows PowerPoint, new 2020 -https://www.acgme.org/Residents-and-Fellows/The-ACGME-for-Residents-and-Fellows

Milestones for Residents and Fellows Flyer, new 2020 https://www.acgme.org/Portals/0/PDFs/Milestones/ResidentFlyer.pdf

Implementation Guidebook, new 2020 - https://www.acgme.org/Portals/0/Milestones%20Implementation%202020.pdf?ver=2020-05-20-152402-013

Assessment Guidebook, new 2020 - https://www.acgme.org/Portals/0/PDFs/Milestones/Guidebooks/AssessmentGuidebook.pdf?ver=2020-11-18-155141-527

Milestones National Report, updated each fall - https://www.acgme.org/Portals/0/PDFs/Milestones/2019MilestonesNationalReportFinal.pdf?ver=2019-09-30-110837-587 (2019)

Milestones Bibliography, updated twice each year - https://www.acgme.org/Portals/0/PDFs/Milestones/MilestonesBibliography.pdf?ver=2020-08-19-153536-447

Developing Faculty Competencies in Assessment courses - https://www.acgme.org/Meetings-and-Educational-Activities/Other-Educational-Activities/Courses-and-Workshops/Developing-Faculty-Competencies-in-Assessment

Assessment Tool: Direct Observation of Clinical Care (DOCC) - https://dl.acgme.org/pages/assessment

Assessment Tool: Teamwork Effectiveness Assessment Module **(TEAM)** - https://dl.acgme.org/pages/assessment

Learn at ACGME has several courses on Assessment and Milestones - https://dl.acgme.org/
